# The Promising Role of Microbiome Therapy on Biomarkers of Inflammation and Oxidative Stress in Type 2 Diabetes: A Systematic and Narrative Review

**DOI:** 10.3389/fnut.2022.906243

**Published:** 2022-05-25

**Authors:** Pradipta Paul, Ridhima Kaul, Basma Abdellatif, Maryam Arabi, Rohit Upadhyay, Reya Saliba, Majda Sebah, Ali Chaari

**Affiliations:** ^1^Division of Medical Education, Weill Cornell Medicine-Qatar, Education City, Qatar Foundation, Doha, Qatar; ^2^Division of Premedical Education, Weill Cornell Medicine-Qatar, Education City, Qatar Foundation, Doha, Qatar; ^3^Department of Medicine—Nephrology and Hypertension, Tulane University, School of Medicine, New Orleans, LA, United States; ^4^Distributed eLibrary, Weill Cornell Medicine-Qatar, Education City, Qatar Foundation, Doha, Qatar

**Keywords:** gut microbiome, gastrointestinal microbiota, clinical trial, inflammatory markers, dysbiosis, resistant dextrin, *Lactobacillus*, *Bifidobacterium*

## Abstract

**Background:**

One in 10 adults suffer from type 2 diabetes (T2D). The role of the gut microbiome, its homeostasis, and dysbiosis has been investigated with success in the pathogenesis as well as treatment of T2D. There is an increasing volume of literature reporting interventions of pro-, pre-, and synbiotics on T2D patients.

**Methods:**

Studies investigating the effect of pro-, pre-, and synbiotics on biomarkers of inflammation and oxidative stress in T2D populations were extracted from databases such as PubMed, Scopus, Web of Science, Embase, and Cochrane from inception to January 2022.

**Results:**

From an initial screening of 5,984 hits, 47 clinical studies were included. Both statistically significant and non-significant results have been compiled, analyzed, and discussed. We have found various promising pro-, pre-, and synbiotic formulations. Of these, multistrain/multispecies probiotics are found to be more effective than monostrain interventions. Additionally, our findings show resistant dextrin to be the most promising prebiotic, followed closely by inulin and oligosaccharides. Finally, we report that synbiotics have shown excellent effect on markers of oxidative stress and antioxidant enzymes. We further discuss the role of metabolites in the resulting effects in biomarkers and ultimately pathogenesis of T2D, bring attention toward the ability of such nutraceuticals to have significant role in COVID-19 therapy, and finally discuss few ongoing clinical trials and prospects.

**Conclusion:**

Current literature of pro-, pre- and synbiotic administration for T2D therapy is promising and shows many significant results with respect to most markers of inflammation and oxidative stress.

## Introduction

Type 2 Diabetes (T2D) is considered an ever-growing burden on public welfare, impacting both high- and low-income nations worldwide. Obesogenic lifestyles, environmental changes, genetic predispositions, and aging have been identified as contributing factors to these increasing trends (1). According to the International Diabetes Federation, as of 2021, an estimated 573 million individuals between 20 and 79 years of age were affected by T2D, representing ∼10% of the world’s population; this figure is expected to cross 643 million by 2030 (2). T2D was responsible for more than 6.7 million deaths in the same year, making it one of the top 10 leading causes of death globally, costing almost 1 trillion USD in health expenditure. In the United States, approximately 21 million adults have been diagnosed with T2D, constituting 8.6% of the adult population (3). Generally, males have a slightly higher prevalence of T2D when compared to females, although this difference is insignificant (3). The onset of new diagnoses increases with increasing age, peaking at around the ages of 55–59 (3).

### Type 2 Diabetes and Coronavirus Disease 2019

The high prevalence of T2D explains its significant role as commonly present comorbidity in patients of the severe acute respiratory syndrome coronavirus–2 (SARS-CoV-2) induced coronavirus disease (COVID-19) that has plagued the globe for two years and counting (4–8). Diabetics are not only increasingly susceptible to contracting the infection, but also have vastly higher mortality associated with the comorbidity, with rates ranging from 10.5% (China) to as high as 33.8% (NYC) and 35.5% (Italy) (9–11). This can be attributed to the compromised nature of the immune system in individuals with type 2 diabetes, where delayed and less effective immune reactions likely lead to longer recovery periods due to higher viral loads (12). The virus is also presumed to thrive in glucose-rich serum in conditions such as those in diabetic patients, since glycolysis induces viral proliferation due to the production of reactive oxygen species (ROS) in the mitochondria and the stimulation of hypoxia-inducible factor 1α (13). Diabetic patients who are infected with COVID-19 are more susceptible to uncontrolled inflammatory responses, thrombophilia, and morbidity (14, 15), although the most commonly presenting symptoms include diarrhea (up to 50%), nausea, vomiting and abdominal pain (16). Researchers elsewhere have recommended modulation of individual diet as a significant factor that should be considered during treatment of the disease given the strict relationship between diet and gut microbiota, and the latter to disease severity (12). It must be also noted, however, that a more pronounced and large-scale effect of the pandemic is the likely reduction of physical activity and healthy diet consumption with respect to not only the general population, but also diabetics, as a result of the lockdown that lags behind the shadow of the virus-mediated effects itself (17).

### Microbiome Therapy in Type 2 Diabetes

The etiology of T2D is complex and is associated with both non-modifiable risk factors such as age, genetic predisposition, race, ethnicity, as well as modifiable factors such as diet, physical activity, apnea and the use of tobacco (18–20). Of these various factors, having poor dietary habits and following a sedentary lifestyle are the two major influences behind the rapidly rising incidence of the diabetes epidemic; corrective measures in one’s lifestyle, including sleep, and diet can help to reduce the risk of onset and prevent or delay the progression of T2D (21–24). Experimental and clinical trials have shown that changes in gut microbiome composition can contribute to T2D pathogenesis; in this regard, pre- and probiotics have closely investigated for the potential to influence the microbiota and thereby promote anti-diabetic activity in a therapeutic manner (25). Probiotics refer to bioactive agents, naturally found in many foods, that administered in adequate amounts, whereas prebiotics refer to substrates utilized by these bioactive agents within the host to grow (26, 27). When provided in combination, they are referred to as synbiotics, and all three are traditionally aimed at improving the quality and quantity of the gastrointestinal microbiome of the host, leading to health benefits. Additionally, a novel category of biologically active substances known as postbiotics, defined as “probiotic-derived products obtained from food-grade microorganisms that confer health benefits when administered in adequate amounts,” have also found to be promising in not only maintaining homeostasis of normal human health, but also for therapeutic purposes in diabetes mellitus (28). The concept of using natural dietary biomolecules, such as non-digestible fibers, flavonoids and other polyphenols, to serve as solutions to clinical challenges is not new, and their promise has been investigated extensively and verified across both literature and by legislative bodies of significance (29–34).

### The Gut Microbiome and Its Dysbiosis

With an astounding composition of 10^11–12^ bacteria per gram in the large intestine, the gut microbiota is an active influence on the adiposity and fat storage capacity of the human body (25). In addition, the gut microbiota regulates the intestinal barrier along with the sensory, immune, neurological, and enteroendocrine systems (35–37). A dysbiosis in the microbiota alters the abundance of species and produces molecules like short chain fatty acids (SCFAs) and lipopolysaccharides (LPS) that affect these systems (26, 37–40). SCFAs, produced by the microbiota during the decomposition of indigestible polysaccharides, improve glucose tolerance by suppressing fat accumulation through binding a G-protein coupled receptor (GPRC) and ultimately increases insulin-sensitivity (38). It has been found that patients with T2D have lower levels of fecal SCFAs than control groups, which can be increased through a daily supplement of inulin-type fructans (41). Additionally, LPS has a high pro-inflammatory potential and drives endotoxemia low-grade inflammation which is suggested to be a potential cause for insulin resistance (38, 39). T2D patients are known to have an altered microbiota (42). Normally, species of the Bacteroidetes and the Firmicutes phyla are the dominant groups of bacteria in the human gut. However, it was found that microbiota from the phylum Firmicutes and class Clostridia are significantly decreased in people with T2D (43). Moreover, the ratios of Bacteroidetes to Firmicutes, and the ratios of Bacteroides-Prevotella group to *Clostridium coccoides*-*Eubacterium rectale* group correlated positively with plasma glucose concentration (43). Decreased proportions of butyrate-producing bacteria and increased proportions of the previously *Lactobacillus* genus species has also been reported (39). Some of the differences observed between the typical microbiota and a T2D-deceased one supports the low-grade inflammation theory suggesting that increased proteobacteria-derived LPS and flagella can induce inflammation (43). A recent review concludes that gut microbiota dysbiosis that promotes inflammation is a general feature of T2D, but a specific signature for diabetes vs. other diseases in terms of biomarkers is not found (39). The review also suggests that the low decrease in α-diversity in T2D patients may explain the low-grade inflammation (39). Gut microbiota modulation in disease and due to biotics involves a multimodal approach; there is competition between various species facilitated by factors such as luminal pH, limited sources, bacterial toxins and SCFAs, in addition to modulation of gut barrier function and promotion of mucus secretion. Moreover, probiotics have been shown to be involved in the differentiation of regulatory T-cells, cytokine modulation and are also important factors in the communication through gut-brain axis level (44).

### Inflammation and Oxidative Stress in Type 2 Diabetes

Chronic activation of the innate immunity, often characterized by cytokine-induced acute inflammation (also called low-grade-inflammation) has been shown to be associated with T2D pathogenesis, in addition to other linked metabolic complications (45). Inflammation has also repeatedly been linked to diabetes pathophysiology through its mediated effects on endothelial and cardiovascular pathology. Characterized by recruitment of leukocytes and their cytokine release, as well as various endothelial and tissue-specific crosstalk, inflammation has shown to play an acute role in metabolic cardiomyopathy (46). Hence, it is not surprising that the elevated presence of pro-inflammatory C-reactive protein (CRP), tumor necrosis factor-α (TNF-α) and interleukin-6 (IL-6) and the reduction of anti-inflammatory cytokines such as IL-4 and IL-10 in the blood are predictive markers of T2D development and pathogenesis (45). Obesity and increased body weight, have been hypothesized to mediate the increased expression of inflammatory markers, leading to downregulation of the intracellular, downstream physiologic effects of insulin, and hence development of further insulin resistance (47). Increased oxidative stress arising from hyperglycemia also impairs glucose uptake in both muscle and fat cells, in addition to decreasing pancreatic β-cell mediated insulin secretion, further contributing to development of diabetes, and serving as an important marker for investigation of therapeutic intervention (48, 49). Whether as a cause or effect, dysbiosis of gut microbiome has been closely associated with T2DM presentation. Such an association could stem from the effect of microbiome dysbiosis on the levels of certain inflammatory markers such as CRP and TFN-α, further resulting in chronic low-grade inflammation following an atypical immune response (50, 51).

### Pro-Inflammatory Markers in Type 2 Diabetes

Pro-inflammatory markers, such as acute-phase proteins, pro-inflammatory cytokines, and chemokines, are elevated in diabetic and obese patients, and are generally found to be at lower levels in individuals with a healthier lifestyle (52). Generally, there is a positive correlation between the levels of pro-inflammatory markers and insulin resistance (53). Several prospective studies have determined that these inflammatory markers may also be used to predict the onset of T2D (54).

C-reactive protein (CRP) is a plasma protein that serves as an early biomarker of inflammation, infection, or trauma (55). CRP is released through IL-6 induced hepatic synthesis and is the main mediator of the acute-phase response. Elevated levels of CRP has a significant association with the risk and onset of Type 2 diabetes, as well as insulin resistance syndrome (56). High concentrations of CRP in patients with T2D may be because of the stimulation of cells in the innate immune system due to increased amounts of nutrients (57). High-sensitivity CRP tests measure lower levels of the protein and is also positively correlated with the risk of type 2 diabetes (58).

Interleukin-6 (IL-6) secreted largely by Th cells, macrophages, fibroblasts, is a multifunctional cytokine involved in inflammatory responses as well as the regulation of cell growth and proliferation, activation, and differentiation of genes. IL-6 induces the production of several leukocytes and proteins such as CRP, and has been found to have parallel associations with T2D (56). The abnormal synthesis of IL-6 causes inflammation and instigates the formation of suppressor of cytokine signaling-3 (SOCS-3), which may serve as an inhibitor of insulin trans-signaling pathways (59). Thus, elevated amounts of this cytokine may serve as a predictor of T2D. Moreover, studies have also shown that IL-6 plays a role in anti-inflammatory processes and glucose metabolism (60).

Another pro-inflammatory cytokine that participates in the regulation of the body’s inflammatory response is Interleukin-1β (IL-1β). IL-β has been associated with several autoimmune diseases, such as Type 1 Diabetes, as well as metabolic syndromes, including cardiovascular diseases and T2D. These disorders are characterized by elevation in IL-1β levels, which lead to weakened secretion of insulin in the pancreas’s B cells (61). IL-1β also causes the breakdown of insulin receptor substrate (IRS) proteins by stimulating suppressors of cytokine signaling (SOCS) (62).

Interferon-gamma is also a pro-inflammatory cytokine that is involved in the preparation of macrophages for stimulation and start of an inflammatory response (63). IFN-γ is expressed by activated T cells and natural killer (NK) cells. In obese patients with T2D, an elevation in IFN-γ can be detected (64). This cytokine has a key role in the maintenance of inflammatory responses in adipose tissues in T2D (65).

Tumor necrosis factor alpha (TNF-α) is a homotrimer that acts as a proinflammatory cytokine and is synthesized by the activation of natural killer (NK) cells, T lymphocytes, and macrophages. It plays critical roles in infection control, bone remodeling, and insulin resistance (66). Obesity and T2D have been associated with increased levels of TNF-α as TNF-alpha instigates insulin resistance in adipose and peripheral tissues through the phosphorylation of serine (67). Furthermore, TNF-α induces low-grade chronic inflammation through the production of ROS and stimulation of several pathways mediated by transcription (68).

Interleukin 17 (IL-17) is a pro-inflammatory T helper (Th) 17 cytokine. IL-17 induces inflammation by binding to a family of IL-17 receptors, which then initiate signaling that activates nuclear factor-Kb (69). This leads to the production of proinflammatory cytokines by monocytes, fibroblasts, and epithelial and endothelial cells (70). IL-17 also moves and employs granulocytes. Insulin resistance and inflammation in diabetes mellitus are linked to the expansion of both Th17 and Th1 (71). Patients with poor blood sugar regulation have elevated serum levels of Th17 cytokines such as IL-17 when compared with individuals with healthy glucose regulation (72).

Interleukin-8 is another proinflammatory chemokine secreted by cells such as adipocytes, macrophages, and endothelial and epithelial cells (73). It is a multifunctional interleukin involved in local and systemic inflammation and macrophage infiltration, and has also been implicated in the pathogenesis of T2D. Increased circulating levels of IL-8 has been reported in patients with T2D (74).

Interleukin-12 (IL-12) is a proinflammatory cytokine that is produced by antigen presenting cells from the innate immune system when in the presence of pathogen-associated molecular patterns (PAMPs) and danger-associated molecular patterns (DAMPs) (75). IL-12 is important for the programming of T cells into Th1 cells, thereby inducing an immune response (76). IL-12, like the other inflammatory cytokines, has been implicated in the pathogenesis of type 2 diabetes and insulin resistance. Levels of IL-12 were found to be the highest in T2D patients (77).

Lipopolysaccharide-binding protein (LBP) is a plasma protein that facilitates the interaction of lipopolysaccharides with other receptors, including toll-like receptor-4 (TLR4) (59). TLR4 activation initiates an intracellular signaling pathway leading to the production of inflammatory cytokines and the activation of the innate immune system (78). Higher concentrations of plasma LPS have been detected in individuals affected by T2D (79).

### Anti-inflammatory Markers in Type 2 Diabetes

Anti-inflammatory markers, such as IL-10, are generally detectable in lower quantities in diabetic and obese patients when compared to controls, and these decreased levels could play a key role in the development of insulin resistance as well as other chronic diseases (80).

Interleukin-10 (IL-10), an anti-inflammatory cytokine, is considered to have a protective function in T2D as it prevents inflammation of immune cells (81). IL-10 is best known for its inhibitory effect on macrophage stimulation (82). Elevated glucose levels in diabetic patients have been found to minimize IL10-mediated activation of STAT3, a signaling protein of IL-10 (81). Thus high serum glucose and HbA1c are associated with low capacity of IL10 production (83).

### Oxidative Stress Markers in Type 2 Diabetes

Oxidative stress is caused by an imbalance of free radicals and antioxidant defenses, and is suggested to have a potential impact on the pathogenesis of diabetes and the development of its complications (84). Values of oxidative stress markers are measured to be mostly higher in diabetic vs. non-diabetic subjects (85).

Superoxide dismutase (SOD) is an oxidative stress-related parameters linked closely to Type 2 diabetes (86). SOD catalyzes the breakdown of reactive oxygen species (ROS) in all tissues and cells (87). Oxidative stress and synthesis of ROS have been associated with the development and complications of diseases such as diabetes mellitus (88). The downregulation of SOD may be associated with the pathogenesis of diabetes mellitus (89). In diabetic patients that have an imbalance of oxidants to antioxidants, there are higher levels of ROS, which causes the total activity of SOD to be significantly higher than the antioxidant’s activity in non-diabetic patients (90). However, the activity of SOD is also linked to the duration of the disease, with the level of antioxidant enzyme decreasing as the years of disease increase (91).

Another antioxidant enzyme is catalase (CAT), found in peroxisomes and the cytosol that mainly functions in the catalysis and disposal of hydrogen peroxide (H_2_O_2_) into O_2_ and H_2_O in erythrocytes (92). Catalase has significantly higher activities in patients T2D when compared with controls (93). Recent data suggests that the onset of diabetes in catalase-deficient patients happens more than 10 years earlier than subjects with normal levels of catalase (94).

Oxidative stress in diabetic patients leads to more severe diabetic complications. Lipid peroxidation results from the interaction of the lipid bilayer of a cell membrane with ROS and oxygen derived free radicals, producing malondialdehyde (MDA) (95, 96). Elevated levels of MDA lead in subjects with T2D to many physiological effects, such as influencing the structural integrity of the cell membrane, inactivating surface enzymes and receptors on the membrane, and causing errors in cell regulation (97).

Nitric oxide (NO) is a reactive nitrogen species (RNS) implicated in the pathogenesis of diabetes and complications. Nitric oxide is involved in impaired cellular function and increased expression of nitric oxide synthase (98). Since nitric oxide has a relatively short half-life, its metabolite nitrite, and nitrate are usually measured in blood and urine, and later used to calculate NO production. Diabetic patients have significantly higher basal levels of NO than non-diabetic individuals across multiple individual studies (99–101), as well as a meta-analysis (102), and were found to have hypertension and microvascular complications. Interestingly, one study based in India showed a lower mean plasma NO level in diabetics compared to control group (103).

Glutathione is the most abundant, low molecular weight non-protein antioxidant produced by cells (104). It exists in the thiol-reduced (GSH) and the disulfide-oxidized (GSSG) forms (105). GSH has a key function in preserving redox homeostasis, transport of amino acids, preventing damage of tissue, and serving as a coenzyme for several reactions (106). As an antioxidant, GSH plays a major role in GSH peroxidase (GPx)—catalyzed reduction of H_2_O_2_, which can in turn be reduced into GSH again by GSH reductase (GR). Because of its function and omnipresence, GSH can also be employed as a biomarker of oxidative stress. More specifically, it has been found that diabetic patients have reduced GSH/GSSG ratios, causing inflammation, hyperlipidemia, and antioxidant imbalance (107, 108).

Oxidative stress exhibits itself in elevated ROS synthesis and oxidation of circulating low-density lipoprotein molecules. Oxidized low-density lipoprotein (oxLDL) activates the immune system and promotes inflammation by inducing dendritic cell maturation and T cell activation (109). The levels of oxLDL were found to be higher in patients with T2D and obesity-related traits relative to controls (110). Other studies have shown that oxLDL levels can predict the onset of the metabolic syndrome (111).

Damage due to oxidative stress can be measured by the amounts of primary or secondary products of peroxidation. One of the secondary byproducts of this reaction are F_2_-isoprostanes (F_2_-IsoP) (112). F_2_-isoprostanes are a group of prostaglandins (PG). F_2_-like products and are involved in many diseases including T2D. Diabetic patients have higher levels of F_2_-IsoP than controls and can be used as a standard biomarker of oxidative stress in patients with T2D (113).

8-hydroxy-2’-deoxyguanosine (8-OHdG) is a modified guanine that is considered a sensitive indicator of oxidative DNA damage (114). A total of 8-OHdG can be used to measure the extent of oxidative stress in the human body as it relates to DNA oxidation ratio and repair of DNA (115, 116). The levels of urine 8-OHdG increases in diabetic patients, and the amount of 8-OHdG generally correlated with the severity of complications such as diabetic nephropathy (117).

Total antioxidant capacity (TAC) is a cumulative measure of small molecule antioxidants and proteins, or the amounts of small molecules alone (118). TAC is often employed to assess a biological sample’s total antioxidant status (TAS). Since the synergistic impact of antioxidants is known to serve greater defense against free radicals than any antioxidant by itself, evaluating overall capacity can provide more insight to the body’s collective mechanisms (119). A negative correlation was observed between HbA1c and plasma TAC and TAS among middle-aged diabetic patients in comparison to healthy controls of similar age (120, 121).

Various factors govern the potential development of T2D interventions using microorganisms to permeate gut microbiota in the host with the aim of targeting biomarkers of inflammation and oxidative stress. Therefore, there is a need in connecting and reporting of the complex data in the literature with aim of distilling T2D interventions using biotics as an effective treatment. Table 1 provides the reference values of each of the above listed biomarkers of inflammation and oxidative stress as seen in controls and those with T2D. 2.

**TABLE 1:** Reference levels of markers of inflammation and oxidative stress in patients with type 2 diabetes and controls.

Marker (units)	T2D	Controls	References
CRP (mg/mL)	3.05 ± 3.92	0.8 ± 1.63	(74)
TNF-a (pg/mL)	8.3 ± 49.9	2.7 ± 4.6	(74)
IL-6 (pg/mL)	9.2 ± 52.1	2.9 ± 4.3	(74)
IL-1β (pg/mL)	30.0 ± 1.2	11.3 ± 1.1	(240)
IL-8 (pg/mL)	69.27 ± 112.8	16.03 ± 24.2	(74)
IL-10 (pg/mL)	9.53 ± 2.27	16.11 ± 2.27	(241)
IL-12 (pg/mL)	147.1± 66.4	69.3 ± 41.6	(77)
IL-17 (pg/mL)	13.32 ± 2.87	5.23 ± 4.18	(242)
SOD (U/gm P/mL)	5.72 ± 0.98	6.67 ± 1.22	(243)
GSH (mg%)	12.20 ± 1.84	14.21 ± 2.55	(243)
GPx (U/gm Hb)	5.92 ± 0.64	8.44 ± 1.17	(243)
GR (U/gm P)	15.24 ± 0.73	16.53 ± 0.41	(243)
CAT (U/gm P/mL)	6.68 ± 0.97	5.79 ± 0.58	(243)
MDA (nmol/mL)	7.09 ± 1.15	4.69 ± 0.72	(243)
F_2_-IsoP (pg/mL)	33.4 ± 4.8	22.2 ± 1.9	(113)
8-OhdG (nmol/mol crea)	2.93 ± 1.78	2.14 ± 0.94	(117)
TAS (mM)	2.19 ± 0.85	1.86 ± 0.65	(244)
Plasma NO_2_^–^ (μ mol/L)	0.21 ± 0.22	0.16 ± 0.19	(101)
Plasma NO_3_^–^ (μ mol/L)	58.5± 42.8	34.5 ± 15.6	(101)
oxLDL-Ab (EU/mL)	26.37 ± 12.86	18.31 ± 8.69	(245)

## Methods

### Literature Sources and Searches

We followed the Preferred Reporting Items for Systematic Reviews and Meta-Analyses (PRISMA) protocol (122). Searches were conducted in PubMed, Scopus, Web of Science, Embase, and Cochrane. We also searched for gray literature through ClinicalTrials.org and ProQuest Dissertations and Theses. The initial search took place in June 2020 and we reran a final search in January 2022 to gather any new records that might have been produced or published.

The search strategy in PubMed included the following elements:

*(“Probiotics”[MeSH Terms] OR “probiotics”[Title/Abstract] OR “probiotic”[Title/Abstract] OR “Prebiotics”[MeSH Terms] OR “prebiotic”[Title/Abstract] OR “prebiotics”[Title/Abstract] OR “Synbiotics”[MeSH Terms] OR “synbiotics”[Title/Abstract] OR “synbiotic”[Title/Abstract] OR “symbiotic”[Title/Abstract] OR “symbiotics”[Title/Abstract] OR “gastrointestinal microbiome”[MeSH Terms] OR “gut microbiome”[Title/Abstract] OR “gut flora”[Title/Abstract]) AND (“diabetes mellitus, type 2”[MeSH Terms] OR “T2D”[Title/Abstract] OR “type 2 diabetes”[Title/Abstract]).*

### Inclusions

Studies had to be clinical trials and randomized studies relevant to the effect of diet using probiotics, prebiotics, and symbiotics on T2D to Prebiotics, Probiotics, and Synbiotics and T2D. Studies of adults of any age, sex, ethnicity, from any region worldwide, and published at any time were included. Further, only those studies reporting on markers of inflammation and/or oxidative stress were included among the final analysis. Reports available from inception of respective databases to accessed dates (final search: January 2022) were included. Covidence was utilized for importing and screening titles, abstracts and full-texts; extraction was performed using MS Excel.

### Exclusions

The paper excluded studies on other type of diabetes, and any reviews, conferences, abstracts and proceedings, editorial and non-clinical papers, as well as animal studies. Studies in other language than English were excluded too. Studies not reporting on markers of inflammation and/or oxidative stress were excluded among the final analysis.

After removing duplicates, the authors independently scanned the title and abstract of all articles referring to the inclusion and exclusion criteria. The same process was also used for full-text screening and any conflicts were resolved by consensus. The included articles were processed for qualitative analysis and the relevant information were grouped by themes and expanded through the discussion.

## Results

### Review Process for Data Extraction

**FIGURE 1:**
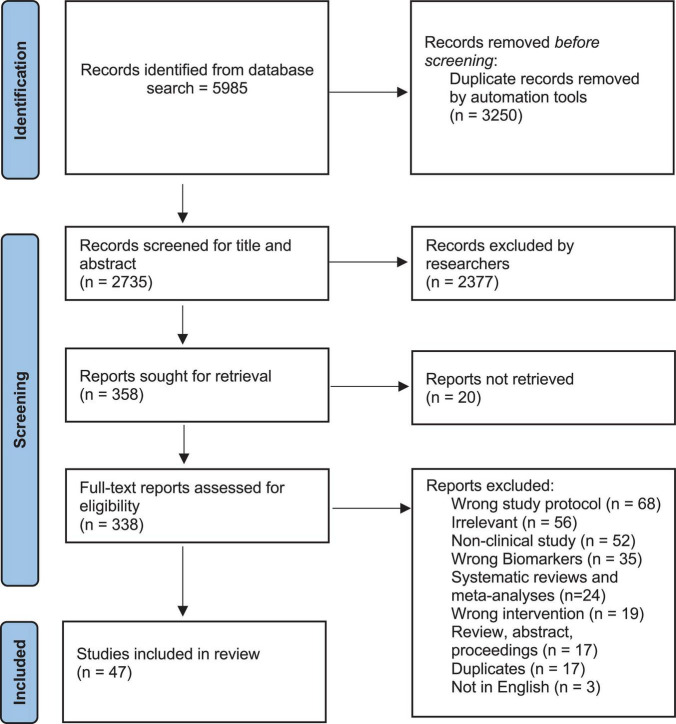
Flowchart of search strategy, inclusion, and exclusion criteria.

According to the PRISMA flowchart (see Figure 1), a total of 5,985 studies were imported from all databases, from which 3,250 duplicates were excluded, while 2,377 articles were found irrelevant through title and abstract screening and 20 others could not be retrieved. Therefore, a total of 338 articles were assessed for eligibility out of which only 47 were included in the final review. The reasons for excluding 311 full-text articles from the study can be summarized as follows: 68 studies did not have the adequate study protocol; 52 studies were non-clinical; 56 studies were not relevant; 35 did not report on the levels of inflammation or oxidative stress; 24 studies were systematic reviews and meta-analysis; 20 studies were not available in full-text; 19 studies had incorrect intervention; 17 studies were reviews, abstract, or proceedings; 17 studies were duplicates; and 3 studies were not in English language.

### Effect of Probiotics on Inflammatory and Oxidative Stress Markers in Type 2 Diabetes

#### Pro-inflammatory Markers in Type 2 Diabetes

##### Effect on C-Reactive Protein and High-Sensitivity C-Reactive Protein

Sixteen studies have investigated and reported the effect of probiotics on either CRP or High-Sensitivity C-Reactive Protein (hs-CRP). While not all studies have reported significant (*p* ≤ 0.05) effects, most have shown that administration of probiotics leads to a general decreasing trend in these markers of inflammation (Table 2).

**TABLE 2:** Studies investigating the effect of probiotics on markers of inflammation and oxidative stress in T2D.

Type of nutraceutical	Study design, country	Participant* demographics size/sex (n, F/M) age (Mean ± SD; yrs.) BMI (Mean ± SD; kg/m^2^)		Control/Placebo substance administered	Interventional nutraceutical administered	Control/Placebo and intervention dose × frequency	Total period of intervention/study	Effect on markers	Mean change in markers Φ	References
		Control/Placebo	Intervention							
Probiotic (Single Sp.)	DB, PC, R (Denmark)	Patients with T2D, IGT or NGT *n* = 24 (24M) 60 (55-66) 28.7 (26.1-31.3)	Patients with T2D, IGT or NGT *n* = 21 (21M) 55 (48-61) 28.1 (25.1-31.1)	1:1 ratio of silicium dioxide and lactose	Freeze-dried *Lactobacillus acidophilus* NCFM capsules (1x10^10^ CFU/g)	1 × 1g/d	4 weeks	TNF-α (§)	0 pg/ml vs +0.1 pg/ml (§)	(132)
								↑IL-6 (§)	+0.1 pg/ml vs +0.2 pg/ml (§)	
								↑IL-1ra (§)	+2 pg/ml vs +31 pg/ml (§)	
								↓CRP (§)	−0.2 mg/l vs +0.4 mg/l (§)	
Probiotic (Single Sp.)	DB, R, PC (Taiwan)	*n* = 22 (13M/9F) 55.77 ± 8.55 27.53 ± 3.15	ADR1 group *n* = 22 (12M/10F) 52.32 ± 10.20 28.04 ± 4.29	NS	Live *Limosilactobacillus reuteri*, ADR-1 (2 × 10^9^ CFU/capsule)	2 × 1 capsules/d	6 months + 3 months follow-up	↓IL-1β (§)	−0.72 ± 1.94 pg/ml vs 0.21 ± 1.52 pg/ml (§)	(134)
								↑CRP (§)	+0.03 ± 0.19 mg/dl vs +0.08 ± 0.28 mg/dl (§)	
								↑IL-6 (§)	+0.95 ± 2.65 ng/ml vs +0.90 ± 1.80 ng/ml (§)	
								↑IL-10 (§)	+1.48 ± 3.09 ng/ml vs +1.04 ± 2.41 ng/ml (§)	
								↑IL-17 (§)	+0.66 ± 3.24 ng/ml vs −0.35 ± 3.63 ng/ml (§)	
								↓TNF-α (§)	−32.00 ± 81.24 pg/ml vs −3.07 ± 72.22 pg/ml (§)	
								↑SOD (§)	+0.93 ± 0.95 U/ml vs +0.39 ± 0.82 U/ml (§)	
								↓GPX (§)	−0.34 ± 3.24 U/ml vs −0.40 ± 3.79 U/ml (§)	
			ADR3 group *n* = 24 (13M/11F) 53.88 ± 7.78 28.03 ± 3.88	NS	Heat-killed *Limosilactobacillus reuteri*, ADR-3 (1 × 10^10^ cells/capsule)	2 × 1 capsules/d	6 months + 3 months follow-up	↓IL-1β	−1.43 ± 2.70 pg/ml vs 0.21 ± 1.52 pg/ml	
								↑CRP (§)	+0.05 ± 0.24 mg/dl vs +0.08 ± 0.28 mg/dl (§)	
								↑IL-6 (§)	+1.55 ± 2.41 pg/ml vs +0.90 ± 1.80 pg/ml (§)	
								↑IL-10 (§)	+2.05 ± 3.25 ng/ml vs +1.04 ± 2.41 ng/ml (§)	
								↑IL-17 (§)	+0.47 ± 2.91 ng/ml vs −0.35 ± 3.63 ng/ml (§)	
								↑TNF-α (§)	+12.81 ± 86.00 pg/ml vs −3.07 ± 72.22 pg/ml (§)	
								↑SOD (§)	+0.04 ± 1.31 U/ml vs +0.39 ± 0.82 U/ml (§)	
								↓GPX (§)	−0.27 ± 4.36 U/ml vs −0.40 ± 3.79 U/ml (§)	
Probiotic (Single Sp.)	R, DB, C, CT (Iran)	Control Group *n* = 27 (12M/15F) 58.2 ± 11.8 BMI NR	Probiotic Group *n* = 30 (10M/20F) 59.7 ± 12.2 29.8 ± 5.7 BMI NR	Capsule containing 0.5 g of rice flour powder	Capsule containing *Lactobacillus acidophilus* (10^8^ CFU)	1 capsule/d	3 months	↑CAT	2.44 ± 0.50 U/ml (I, 3m) vs 1.95 ± 0.34 U/ml (C, 3m)	(144)
								↑GPX	92.15 ± 8.41 U/mL (I, 3m) vs 84.89 ± 6.52 U/mL (C, 3m)	
								↑SOD	4.58 ± 0.42 U/mL (I, 3m) vs 3.99 ± 0.27 U/mL (C, 3m)	
								↓OxLDL (§)	16.85 ± 1.53 mU/L (I, 3m) vs 17.07 ± 1.01 mU/L (C, 3m) (§)	
Probiotic (Single Sp.)	R, DB, C, CT (Iran)	Control Bread (CB) *n* = 27 (5M/22F) 53.4 ± 7.5 30.5 ± 4.1	Probiotic Bread *n* = 27 (5M/22F) 52.0 ± 7.2 29.8 ± 5.7	Control bread	Bread containing Bacillus coagulans (1 × 10^8^ CFU/g)	40 × 3 g/d	8 weeks	↓hs-CRP (§)	−1,330.2 ± 2,924.1 ng/ml vs −586.9 ± 2,009.2 ng/ml (§)	(160)
Probiotic (Single sp.)	DB, R, PG, PC (Sweden)	T2D and obese patients* n = 15 (11M/4F) 65 ± 5 30.7 ± 4.0	T2D and obese patients*; Low Dose group *n* = 15 (12M/3F) 66 ± 6 30.6 ± 4.5	Capsule with mildly sweet tasting powder in an aluminum laminate stick pack	Capsule containing low-dose *Limosilactobacillus reuteri* DSM 17938 (10^8^ CFU/capsule)	1 capsule/d	12 weeks	hs-CRP (§)	2.3 ± 2.8 mg/L (I, 12w) vs 2.3 ± 2.8 mg/L (I, B) (§)	(246)
			T2D and obese patients*; High dose group *n* = 14 (11M/3F) 64 ± 6 32.3 ± 3.4	Capsule with mildly sweet tasting powder in an aluminum laminate stick pack	Capsule containing high-dose *Limosilactobacillus reuteri* DSM 17938 (10^10^ CFU/capsule)	1 capsule/d	12 weeks	↑hs-CRP (§)	2.4 ± 2.1 mg/L (I, 12w) vs 2.0 ± 1.4 mg/L (I, B) (§)	
Probiotic (Single Sp.)	PG, DB, RCT (Iran)	T2D patients with nephropathy* n = 20 (10M/10F) 53.6 ± 1.6 26.58 ± 0.73	T2D patients with nephropathy* n = 20 (9M/11F) 56.90 ± 1.81 26.68 ± 0.71	Conventional soy milk	Probiotic soy milk containing Lactiplantibacillus plantarum A7 (2 × 10^7^ CFU/mL)	200 mL/d	8 weeks	↑(x)TNF-α (§)	172.83 ± 7.6 pg/ml (I, 8w) vs 172.44 ± 5.7 pg/ml (I, B) (§)	(133)
								↓CRP (§)	4.2 ± 1.4 mg/L (I, 8w) vs 4.5 ± 1.9 mg/L (I, B) (§)	
Probiotic (Single Sp.)	PG, DB, RCT (Iran)	T2D patients with nephropathy* n = 20 (10M/10F) 53.6 ± 1.6 26.58 ± 0.73	T2D patients with nephropathy* n = 20 (9M/11F) 56.90 ± 1.81 26.68 ± 0.71	Conventional soy milk	Probiotic soy milk containing Lactiplantibacillus plantarum A7 (2 × 10^7^ CFU/mL)	200 mL/d	8 weeks	↓MDA (§)	1.28 ± 0.11 μmol/L (I, 8w) vs. 1.35 ± 0.05 μmol/L (I, B) (§)	(141)
								↓MDA	Significant reduction vs control, markers NS	
								↑TAC (§)	989.06 ± 30.25 mmol/L (I, 8w) vs. 960.06 ± 35.20 mmol/L (I, B) (§)	
								↑GSH	732.96 ± 61.95 μmol/L (I, 8w) vs. 600.66 ± 69.61 μmol/L (I, B);	
									also significant MD vs control, markers NS	
								↓GSSG	19.00 ± 0.70 μmol/L (I, 8w) vs. 30.37 ± 0.20 μmol/L (I, B);	
									also significant MD vs control, markers NS	
								↑GPX	1.30 ± 1.25 U/g Hb (I, 8w) vs. 0.87 ± 1.00 U/g Hb (I, B);	
									also significant MD vs control, markers NS	
								↑GR	1.35 ± 0.05 U/g Hb (I, 8w) vs. 0.97 ± 1.02 U/g Hb (I, B);	
									also significant MD vs control, markers NS	
Probiotic (Single Sp.)	R, DB, PC (Denmark)	*n* = 18 (18M) 60.6 ± 5.2 27.7 ± 3.3	*n* = 23 (23M) 58.5 ± 7.7 29.2 ± 3.8	Artificially acidified milk	”Cardi04” yogurt containing *Lactobacillus helveticus*	300 × 1 mL/d	3 months	↑(x) hsCRP (§)	0.6 (0.4; 1.6) mg/L (I, 3m) vs 0.7 (0.4; 2.1) mg/L (I, B) (§)	(247)
								↑TNF-α (§)	1.2 ± 0.4 pg/ml (I, 3m) vs 1.1 ± 0.3 pg/ml (I, B) (§)	
Probiotic (Single Sp.)	R, DB, PC, CT (Iran)	Control Bread (CB) *n* = 27 (5M/22F) 53.4 ± 7.5 30.5 ± 4.1	Probiotic Bread *n* = 27 (5M/22F) 52.0 ± 7.2 29.8 ± 5.7	Control bread	Bread containing Bacillus coagulans (1 × 10^8^ CFU/g)	40 × 3 g/d	8 weeks	↑NO (§)	+18.5 ± 36.2 μmol/L vs −0.8 ± 24.5 μmol/L (§)	(142)
								↑TAC (§)	+78.6 ± 218.4 mmol/L vs. −45.7 ± 240.3 mmol/L (§)	
								↑GSH (§)	+6.2 ± 347.2 μmol/L vs +18.8 ± 417.8 μmol/L	
								↑CAT (§)	+4.1 ± 20.2 U/mL vs +2.7 ± 14.9 U/mL	
								↑MDA (§)	+0.6 ± 1.7 μmol/L vs +0.5 ± 1.5 μmol/L (§)	
Probiotic (Single sp.)	PG, DB, RCT (Iran)	T2D patients with nephropathy* n = 20 (10M/10F) 53.6 ± 1.6 26.58 ± 0.73	T2D patients with nephropathy* n = 20 (9M/11F) 56.90 ± 1.81 26.68 ± 0.71	Conventional soy milk	Probiotic soy milk containing Lactiplantibacillus plantarum A7 (2 × 10^7^ CFU/mL)	200 mL/d	8 weeks	↑SOD	Markers NS	(147)
								↓8-OHdG	Markers NS	
Probiotic (Single sp.)	R, PC (Japan)	*n* = 34 (20M/14F) 65.0 ± 8.3 24.6 ± 2.6	*n* = 34 (29M/5F) 64.0 ± 9.2 24.2 ± 2.6	Fermented milk without probiotics	Lacticaseibacillus casei strain Shirota-fermented milk (> 4 × 10^10^ cells per bottle)	80 mL/d	16 weeks	↑hs-CRP	+92.0 mg/dL vs +32.5 mg/dL	(135)
								^.^TNF-α (§)	+0.0 ± 0.5 pg/ml vs +0.0 ± 0.3 pg/ml (§)	
								↑IL-6 (§)	+0.4 ± 2.8 pg/ml vs +0.2 ± 1.0 pg/ml (§)	
								^.^LBP (§)	+0.0 ± 3.8 μg/mL vs −0.5 ± 4.0 μg/mL (§)	
Probiotic (Multi sp.)	DB, PC, RCT (Saudi Arabia)	*n* = 30 (NS) 46.6 ± 5.9 30.1 ± 5.0	*n* = 31 (NS) 48.0 ± 8.3 29.4 ± 5.2	Freeze-dried maize starch and maltodextrins	EcologicBarrier (2.5 × 10^9^ CFU/g; Bifidobacterium bifidum W23, *Bifidobacterium animalis* subsp. lactisW52, *Lactobacillus acidophilus* W37, *Levilactobacillus brevis*W63, *Lacticaseibacillus casei* W56, *Ligilactobacillus salivarius* W24, *Lactococcus lactis* W19 and *Lactococcus lactis* W58) with maize starch and maltodextrins	2 × 2 g/d	6 months	↓IL-6	−3.9 pg/ml (−76.5%)	(123)
								↓IL-6 (§)	−3.9 pg/ml (−76.5%) vs −2.8 pg/ml (−77.8%) (§)	
								↓TNF-α	−0.6 pg/ml or −66.7%	
								↓TNF-α (§)	−0.6 pg/ml (−66.7%) vs −0.2 (−40.0%) (§)	
								↓CRP	−2.9 μg/ml or −52.7%	
								↓CRP (§)	−2.9 μg/ml (−52.7%) vs +0.4 μg/ml (+13.3%) (§)	
								↓Endotoxin	−3.2 IU/ml or −69.6%	
								↓Endotoxin (§)	−3.2 IU/ml (−69.6%) vs +0.8 IU/ml (+38.1%) (§)	
Probiotic (Multi sp.)	DB, PC, RCT (Saudi Arabia)	*n* = 39 (21M/18F) 46.6 ± 5.9 30.1 ± 5.0	*n* = 39 (19M/20F) 48.0 ± 8.3 29.4 ± 5.2	Maize starch and maltodextrins	EcologicBarrier (2.5 × 10^9^ CFU/g) containing *Bifidobacterium bifidum* W23, *Bifidobacterium animalis* subsp. *lactis*W52, *Lactobacillus acidophilus* W37, *Levilactobacillus brevis*W63, *Lacticaseibacillus casei* W56, *Ligilactobacillus salivarius* W24, *Lactococcus lactis* W19 and *Lactococcus lactis* W58 with maize starch and maltodextrins	2 × 2 g/d	3 months	↓Endotoxin	−2.40 IU/mL (−52.2%) vs −0.20 (−9.5%) IU/mL	(140)
								↑Endotoxin (§)	+0.15 IU/mL (§)	
Probiotic (Multi sp.)	DB, R, C, CT (Iran)	*n* = 30 (12M/18F) 51.00 ± 7.32 29.14 ± 4.30	*n* = 30 (11M/19F) 50.87 ± 7.68 28.95 ± 3.65	Conventional yoghurt containing Lactobacillus delbrueckii subsp. bulgaricus, Streptococcus thermophilus	Probiotic yoghurt containing *Lactobacillus delbrueckii* subsp. *bulgaricus*, *Streptococcus thermophilus*, *Bifidobacterium animalis* subsp. *lactis* Bb12 (1.79–6.04 × 10^6^ CFU/g) and *Lactobacillus acidophilus* La5 (1.85–7.23 × 10^6^ CFU/g)	300 g/d	6 weeks	↑Erythrocyte SOD	1113.69 ± 177.77 U/g Hb (I, 6w) vs 975.80 ± 238.34 U/g Hb (I, B);	
									significant MD vs control, markers NS	(143)
								↑GPX	29.81 ± 4.58 U/g Hb (I, 6w) vs 29.03 ± 4.29 U/g Hb (I, B);	
									significant MD vs control, markers NS	
								↓CAT (§)	146.57 ± 34.05 K/g Hb (I, 6w) vs 148.81 ± 34.56 K/g Hb (I, B) (§)	
								↑TAS	0.96 ± 0.18 mmol/L (I, 6w) vs 0.90 ± 0.18 mmol/L (I, B);	
									significant MD vs control, markers NS	
								↓MDA	2.53 ± 0.65 μmol/L (I, 6w) vs 2.79 ± 0.62 μmol/L (I, B)	
Probiotic (Multi sp.)	DB, R, C, CT (Iran)	T2D and overweight patients* n (I+C) = 42 (10M/32F) 49.00 ± 7.08 29.22 ± 3.20	T2D and overweight patients* n (I+C) = 42 (10M/32F) 53.00 ± 5.9 28.36 ± 4.14	Conventional yoghurt containing Lactobacillus delbrueckii subsp. bulgaricus and Streptococcus thermophilus	Probiotic yoghurt containing *Lactobacillus delbrueckii* subsp. *bulgaricus*, *Streptococcus thermophilus*, *Bifidobacterium animalis* subsp. *lactis* Bb12 (∼3.7 × 10^6^ CFU/g) and *Lactobacillus acidophilus* La5 (∼3.7 × 10^6^ CFU/g)	300 g/d	8 weeks	↓hs-CRP (§)	2.80 ± 1.48 mg/l (I, 8w) vs 3.26 ± 1.36 mg/l (I, B) (§)	(129)
								↓IL-6 (§)	22.18 ± 2.56 pg/ml (I, 8w) vs 22.60 ± 2.81 (I, B) (§)	
								↓TNF-α	2.92 ± 1.16 pg/ml (I, 8w) vs 4.36 ± 1.9 pg/ml (I, B);	
									also significant MD vs control, markers NS	
Probiotic (Multi sp.)	SB, CT (Iran)	*n* = 18 (I+C = 8M/26F) 51.8 ± 10.2 27.24 ± 2.73	*n* = 16 (I+C = 8M/26F) 55.4 ± 8 27.97 ± 3.81	1000g Magnesium stearate/1500 mg capsule	*Lactobacillus acidophilus*, *L. bulgaricus, L. bifidum and L. casei*	2 × 1500 mg/d	6 weeks	↓MDA (§)	4.24 ± 0.44 μmol/L (I, 6w) vs 5.09 ± 0.53 μmol/L (I, B) (§)	(128)
								↓IL-6 (§)	3.83 ± 0.35 pg/ml (I, 6w) vs 4.51 ± 0.45 pg/ml (I, B) (§)	
								↑hs-CRP (§)	4333.81 ± 1256.6 ng/ml (I, 6w) vs 3174.87 ± 701.77 ng/ml (I, B) (§)	
Probiotic (Multi sp.)	R, DB, PC, CT (Iran)	*n* = 27 (Sex NS) 52.59 ± 7.14 30.17 ± 4.23	*n* = 27 (Sex NS) 50.51 ± 9.82 31.61 ± 6.36	100 mg fructo-oligosaccharide with lactose/capsule	Freeze-dried *Lactobacillus acidophilus* (2 × 10^9^ CFU), L. casei (7 × 10^9^ CFU), *L. rhamnosus* (1.5 × 10^9^ CFU), *L. bulgaricus* (2 × 10^8^ CFU), *Bifidobacterium breve* (2 × 10^10^ CFU), *B. longum* (7 × 10^9^ CFU), *Streptococcus thermophilus* (1.5 × 10^9^CFU), and 100 mg fructo-oligosaccharide with lactose/capsule	1 capsule/d	8 weeks	↓hs-CRP	−777.57 ± 441.7 ng/ml vs +878.72 ± 586.44 ng/ml	(124)
								↑TAC (§)	+379.97 ± 41.8 mmol/L vs. +84.94 ± 24.32 mmol/L (§)	
								↑GSH	+240.63 ± 101.29 μmol/l vs −33.46 ± 69.54 μmol/l	
Probiotic (Multi sp.)	R, DB, PG, PC (Brazil)	*n* = 22 (14M/8F) 50.95 ± 7.20 27.94 ± 4.15	*n* = 23 (12M/11F) 51.83 ± 6.64 27.49 ± 3.97	Conventional fermented goat milk with Streptococcus thermophilus TA-40	Probiotic fermented goat milk with *Lactobacillus acidophilus* La-5 (1.62-77.2 × 10^6^ CFU/g) and *Bifidobacterium animalis* subsp. *lactis* BB-12 (1.56–44.5 × 10^7^ CFU/g)	120 g/d	6 weeks	↑TAS (§)	+0.01 mM (−0.07 to +0.08) (§)	(146)
								↓F2-iso (§)	−2.59 pg/ml (−21.41 to 16.23) (§)	
Probiotic (Multi sp.)	DB, PC, PG, RCT (Ukraine)	*n* = 26 (NR) 55.73 ± 8.76 35.63 ± 7.76	*n* = 28 (NR) 56.29 ± 11.14 35.66 ± 5.35	Organoleptically similar formulation as intervention	Multiprobiotic symbiter forte omega combination of *Lactobacillus* (1.0 × 10^9^ CFU/g), *Bifidobacterium* (1.0 × 10^9^ CFU/g), *Lactococcus* (1.0 × 10^8^ CFU/g), *Propionibacterium* (1.0 × 10^8^ CFU/g), Acetobacter (1.0 × ^10^ CFU/g), 2.0% bentonite, 3.0% wheat germ oil feed, 2.5% flax seed oil and, 2.5% wheat germ with 0.5–5% omega-3	10 × 1 g/d	8 weeks	↓TNF-α	−5.81 ± 9.13 pg/ml vs +0.38 ± 9.05 pg/ml	(138)
								↓IL-1β	−4.1 ± 8.36 pg/ml	
								↓IL-1β (§)	−4.1 ± 8.36 pg/ml vs −1.67 ± 7.20 pg/ml (§)	
								↓IL-6	−6.77 ± 9.62 pg/ml	
								↓IL-6 (§)	−6.77 ± 9.62 pg/ml vs −4.70 ± 11.38 pg/ml (§)	
								↓ IL-8	−8.99 ± 21.11 pg/ml	
								↓ IL-8 (§)	−8.99 ± 21.11 pg/ml vs −1.83 ± 33.78 pg/ml (§)	
								↓ IFN-λ (§)	−3.11 ± 39.94 pg/ml vs +7.79 ± 40.63 pg/ml (§)	
Probiotic (Multi sp.)	DB, PC, PG, RCT (Ukraine)	Patients with T2D and NAFLD *n* = 24 (NR) 57.38 ± 9.92 32.55 ± 3.62	Patients with T2D and NAFLD *n* = 26 (NR) 53.23 ± 10.09 33.19 ± 4.93	Organoleptically similar formulation as intervention	Symbiter forte (combination of 250 mg smectite gel and *Bifidobacterium* (1 × 10^10^ CFU/g), *Lactobacillus* + *Lactococcus* (6 × 10^10^ CFU/g), acetobacter (1 × 10^6^ CFU/g) and SCFAs producing *Propionibacterium* (3 × 10^10^ CFU/g) genera)	10 × 1 g/d	8 weeks	↓ IL-1β	−6.74 ± 15.59 pg/ml or −6.00 ± 33.0 %	(139)
								↓TNF-α	−6.75 ± 7.73 pg/ml or −12.17 ± 14.4 %	
Probiotic (Multi sp.)	SC, DB, PC, PG, RCT (Ukraine)	*n* = 22 (NR) 57.18 ± 2.06 35.65 ± 1.57	*n* = 31 (NR) 52.23 ± 1.74 34.70 ± 1.29	Organoleptically similar formulation as intervention	Multiprobiotic “Symbiter” combination of *Lactobacillus* + *Lactococcus* (6 × 10^10^ CFU/g), *Bifidobacterium* (1.0 × 10^10^ CFU/g), *Propionibacterium* (3 × 10^10^ CFU/g), *Acetobacter* (1.0 × 10^6^ CFU/g)	10 × 1 g/d	8 weeks	↓TNF-α	−7.95 ± 1.27 pg/ml vs +1.03 ± 2.07 pg/ml	(136)
								↓IL-1β	−5.44 ± 1.51 pg/ml vs +0.45 ± 1.97 pg/ml	
								↓IL-6	−3.45 ± 1.48 pg/ml	
								↓IL-6 (§)	−3.45 ± 1.48 pg/ml vs −1.89 ± 1.28 pg/ml (§)	
								↓ IL-8	−3.80 ± 1.05 pg/ml	
								↓ IL-8 (§)	−3.80 ± 1.05 pg/ml vs −3.85 ± 1.66 pg/ml (§)	
								↓ IFN-λ (§)	−13.80 ± 7.04 pg/ml vs +6.16 ± 8.88 pg/ml (§)	
Probiotic (Multi sp.)	PC, DB, RCT (Ukraine)	*n* = 27 (Sex NR) 56.93 ± 9.88 32.28 ± 6.08	*n* = 28 (Sex NR) 53.82 ± 9.58 31.99 ± 6.02	Organoleptically similar formulation as intervention	Symbiter forte (combination of 250 mg smectite gel and *Bifidobacterium* (1 × 10^9^ CFU/g), *Lactobacillus* (1 × 10^9^ CFU/g), *Lactococcus* (1 × 10^8^ CFU/g), *Acetobacter* (1 × 10^5^ CFU/g) and *Propionibacterium* (1 × 10^8^ CFU/g) genera	10 × 1 g/d	8 weeks	↓ IL-1β (§)	−4.91 ± 8.23 pg/ml vs −0.41 ± 9.61 pg/ml (§)	(137)
								↓ IL-1β	−4.91 ± 8.23 pg/ml	
								↓TNF-α	−5.02 ± 9.33 pg/ml vs +0.69 ± 10.01 pg/ml	
								↓IL-6	−4.11 ± 7.15 pg/ml vs −0.70 ± 5.80 pg/ml	
								↓ IL-8 (§)	−5.6 ± 13.92 pg/ml vs −2.16 ± 11.49 pg/ml (§)	
								↓ IL-8	−5.6 ± 13.92 pg/ml	
Probiotic (Multi sp.)	DB, R, PG, PC (Malaysia)	*n* = 68 (34M/34F) 54.2 ± 8.3 29.3 ± 5.3 *n* = 53 (PP analysis)	*n* = 68 (31M/37F) 52.9 ± 9.2 29.2 ± 5.6 *n* = 47 (PP analysis)	Organoleptically similar sachets without probiotic	Sachets containing viable microbial cell preparation of *Lactobacillus acidophilus*, *L. casei*, *L. lactis*, *Bifdobacterium bifdum*, *B. longum* and *B. infantis* (0.5 × 10^10^ CFU, each) in 250 mL water	2 sachets/d	12 weeks	↑hs-CRP (§)	+0.23 ± 2.7 mg/L vs −0.36 ± 3.0 mg/L (§)	(130)
Probiotic (Multi sp.)	R, DB, PC (Iran)	T2D and CHD patients* n = 27 (10M/17F)) 62.4 ± 13.1 29.9 ± 5.0	T2D and CHD patients* n = 27 (11M/16F) 64.8 ± 8.3 31.4 ± 5.8	”Barij Essence”	LactoCare containing *Lactobacillus acidophilus*, *L. reuteri*, *Limosilactobacillus fermentum* and *Bifidobacterium bifidum* (2 × 10^9^ CFU/g each) and 200 μg/d selenium yeast	1/d	3 months	↑NO	+7.86 μmol/L	(125)
								↑GSH	+154.16 μmol/L	
								↑TAC	+119.30 mmol/L	
								↓hs-CRP	−1043.28 ng/mL	
								↑MDA (§)	+0.10 μmol/L (§)	
Probiotic (Multi sp.)	R, DB, PC (Iran)	Patients with T2D and CHD* n = 30 (Sex NS) 61.8 ± 9.8 29.3 ± 4.1	Patients with T2D and CHD* n = 27 (Sex NS) 60.7 ± 9.4 30.3 ± 5.2	NS	Supplements containing *Bifdobacterium bifdum* (2 × 10^9^ CFU/d), *L. casei* (2 × 10^9^ CFU/d), *Lactobacillus acidophilus* (2 × 10^9^ CFU/d)	1/d	3 months	↑NO	+4.28 μmol/L	(126)
								↑GSH	+45.15 μmol/L	
								↑TAC	+108.44 mmol/L	
								↓hs-CRP	−0.88 mg/L	
								↓MDA (§)	−0.23 μmol/L (§)	
Probiotic (Multi sp.)	R, DB, PC (Iran)	Patients with T2D and CHD* n = 30 (14M/16F) 67.3 ± 11.0 28.2 ± 4.9	Patients with T2D and CHD* n = 30 (16M/14F) 71.5 ± 10.9 29.0 ± 6.2	NS	50,000 IU vitamin D3 every 2 weeks and *Lactobacillus acidophilus*, *L. reuteri*, *Limosilactobacillus fermentum* and *Bifidobacterium bifidum* (each 2 × 10^9^ CFU/g)	1/d	12 weeks	↑NO	+1.7 ± 4.0 μmol/L vs −1.4 ± 6.7 μmol/L	(127)
								↑GSH (§)	+18.0 ± 112.7 μmol/L vs −12.2 ± 122.5 μmol/L (§)	
								↑TAC	+12.6 ± 41.6 mmol/L vs −116.9 ± 324.2 mmol/L	
								↓hs-CRP	−950.0 ± 1811.2 ng/mL vs +260.5 ± 2298.2 ng/mL	
								↓MDA	−0.1 ± 0.3 μmol/L vs +0.1 ± 0.7 μmol/L	
Probiotic (Sp. NS)	PG, R, CT (Iran)	(C1) C. ficifolia group *n* = 20 (12M/8F) 51.8 ± 2.24 28.95 ± 3.34 (C2) Dietary advice group *n* = 20 (9M/11F) 46.95 ± 9.34 29.75 ± 4.66	Probiotic yogurt group *n* = 20 (3M/17F) 54.1 ± 9.54 28.77 ± 4.59	(1) C. ficifolia (2) Dietary Advice	Probiotic (species NS) yogurt	(C1) 100 × 1 g/d (C2) NS (I) 150 × 1 g/d	8 weeks	↓hs-CRP	1.13 ± 0.29 mg/L (I, 2w) vs 1.29 ± 0.27 mg/L (I, B);	
									significant vs dietary advice group, markers NS	(131)
					Probiotic (species NS) yogurt and *C. ficifolia*	150 × 1 g/d probiotic yogurt, 100 × 1 g/d *C. ficifolia*	8 weeks	↓hs-CRP	1.13 ± 0.34 mg/L (I, 2w) vs 1.69 ± 0.25 mg/L (I, B);	
									significant vs dietary advice group, markers NS	

*All participants are T2D-diagnosed patients, unless otherwise stated; ↓ indicates a decrease in value; ↑ indicates an increase in value; ^.^ = No change in value; ↑(x) = indicates prevention of increase in value compared to control; Φ = Order of markers compared = those of Intervention (I) group first, Control (C) or baseline (B) second; Text color represents comparison body as follows, Blue = Comparison of effective change due to intervention by adjusted Mean Difference (MD) of changes in markers observed between I&C groups at end of the study from baseline; Green = Comparison of changes in I vs. C groups at the end of study from respective baselines; Red = Comparison of change (or difference) in markers at the end of study from baseline in I group; § = Non-significant Result; T2D = Type-2 Diabetes; NS = Not Specified; NR = Not Reported; Sp. = Species; SB = Single-Blinded; DB = Double-Blinded; R = Randomized; RCT = Randomized Controlled Trial; CC = Crossover Controlled; PC = Placebo-Controlled; PG = Parallel Group; CT = Clinical Trial;; OL = Open Label; MDA = Malondialdehyde; IL-6 = Interleukin 6; TNF-α = Tumor necrosis factor alpha; CRP = C-reactive protein; SCFA = Short-Chain Fatty Acid; NAFLD = Non-Alcoholic Fatty Liver Disease; GSH: Glutathione; GSSG = oxidized glutathione; Hs-CRP: High sensitivity C-Reactive Protein; CAT = Catalase; TAC = Total Antioxidant Capacity; TAS = Total Antioxidant Status; LPS = lipopolysaccharide; LBP = lipopolysaccharide binding protein; IL-1ra = IL-1 receptor antagonist; SOD = Superoxide Dismutase; GPX = Glutathione Peroxidase; GR = glutathione reductase; HP = High Performance;; F2-iso = F2-isoprostane; IFG = impaired fasting glucose; IGT = impaired glucose tolerance; NGT = normal glucose tolerance; IGT = impaired glucose tolerance; CHD = Coronary Heart Disease.

Sabico et al. (123) reported a significant decrease of 2.9 mg/L (-52.7%) in CRP following supplementation of a multi-species probiotic in a cohort of 31 T2D patients when compared to baseline values; however, these results were not found to be significant compared to control despite an increase of 0.4 mg/L (+ 13.3%) in CRP markers in this group. Another group of researchers from Iran reported a significant decrease of 0.777 ± 0.441 mg/L of hs-CRP following an 8-week intervention of probiotics compared to a control group (124). Similar significant results have been reported following 12-week multi-species probiotic supplementation in three cohorts of T2D patients with coronary heart disease (CHD), with adjusted mean differences of –1.04, –0.88, and –0.95 mg/L in hs-CRP compared to control (125–127). Three other studies investigating the effects of multi-species produced insignificant results, with two reporting slight increases and another reporting a decrease in hs-CRP compared to baseline values or that of control (128–130). Of interest, Bayat et al. (131) have shown that an 8-week probiotic of an unknown number of species administration with and without *Cucurbita ficifolia* resulted in significant reductions in serum hs-CRP compared to baseline, control, and another receiving only *C. ficifolia*.

The results of single-species probiotic supplementation on CRP and hs-CRP appear to be non-conclusive, with independent studies reporting largely non-significant (*p* > 0.05) mean increase and decrease in these markers in the intervention groups compared to control or baseline. Andreasen et al. (132) and Feizollahzadeh et al. (133) have reported mean decreases of 0.3 and 0.2 mg/L in CRP compared to baseline following supplementation with soymilk containing *Lactiplantibacillus plantarum* (previously *Lactobacillus plantarum)* and freeze-dried *Lactobacillus acidophilus* in T2D-associated nephropathic patients and a mixed group of diabetic or those with impaired or normal glucose tolerance, respectively. Hsieh et al. (134) have reported that consuming live and heat-killed *Limosilactobacillus reuteri* (previously *Lactobacillus reuteri*) resulted in an increase of 0.3 ± 1.9 and 0.5 ± 2.4 mg/L from baseline(s), respectively, in two cohorts of T2D patients without any specified/reported comorbidities; although, since these results were all non-significant, no solid conclusion can be made regarding the effect of the difference in probiotic species administered or the presence of diagnosed comorbidities. Similar conflicting and non-significant results have been shown for changes in hs-CRP following single-probiotic supplementation, while one study by Sato et al. (135) have shown that Shirota-fermented milk with *Lacticaseibacillus casei* (previously *Lactobacillus casei*) significantly increased detectable hs-CRP levels by 92.0 mg/dL compared to an increase of 32.5 mg/dL in the control group over a period of 16 weeks.

##### Effect on Tumor Necrosis Factor-α

Eleven studies have investigated and reported the effect of probiotics either single or multi-species, on TNF-α. While some studies have reported non-significant (*p* > 0.05) changes post probiotic supplementation, most trials have successfully concluded that administration of probiotics lead to significant overall decreases in levels of this pro-inflammatory marker (Table 2).

Sabico et al. (123) reported that a multi-species probiotic supplementation led to a 0.6 pg/mL or 66.7% decrease from baseline in a cohort of diabetic patients from Saudi Arabia. Similarly, another group reported a near 33% reduction in the marker from baseline, a change that was also found to be significant compared to control (129). Through a series of four studies, Kobyliak et al. (136–139) have reported significant changes of –5.81 ± 9.13, –7.95 ± 1.27, –5.02 ± 9.33, and –6.75 ± 7.73 pg/mL in TNF- α from baseline compared to control following multi-species probiotic supplementation.

On the other hand, there is no consensual trend among studies investigating the effect of single-species probiotics due to the lack of statistically significant observations. However, interestingly, *L. reuteri* supplementation produced a directionally different change depending on whether the bacteria was provided in heat-killed or live form (134). Other studies have reported little to no change in TNF-α following single-species probiotic administration (132, 133, 135).

##### Effect on Interleukin-1β

Information for the effect of single or multi species probiotics on IL-1β is available from five clinical studies, each reporting significant results of decrease in marker compared to either baseline or control in the intervention groups (Table 2).

Hsieh et al. (134) have demonstrated that supplementation with heat-killed *L. reuteri* over a course of 6 months lead to a significant decrease of 1.43 ± 2.70 pg/mL in IL-1β compared to the change in control group over the same period. Interestingly, supplementation of the live form of the same bacteria in a lower dose resulted in a 50% reduction in this change from baseline and was not significant compared to control in the same study.

The effect of multi-species probiotic supplementation has been more consistently reproduced across multiple studies by Kobyliak et al. (136–139). Based on multispecies probiotic “Symbiter Forte” supplementation across four cohorts of diabetics, the group has reported significant mean differences of –4.1 ± 8.36, –4.91 ± 8.23, –6.74 ± 15.59, and –5.44 ± 1.51 pg/mL in serum IL-1β from baseline, with the latter two results also reported to be statistically significant compared to effects of placebos with organoleptically similar formulation as the probiotic media.

##### Effect on Interleukin-6

Probiotic supplementation has been investigated among diabetics in a total of nine studies, including both single and multi-species probiotics; results were found to be inconclusive with reporting of both positive and negative, significant, and insignificant findings, across multiple studies (Table 2).

Across three single-species probiotic administering studies, organisms of the (previously) *Lactobacillus* genus have been associated with slight, statistically non-significant increases in IL-6. These range from average changes of + 0.1 and + 0.4 pg/mL levels following 1 and 4 months of intervention, respectively, to as high as + 0.95 and + 1.55 pg/mL following 6 months of supplementation and 3 months of follow-up (132, 134, 135).

On the other, hand, the six studies investigating the change in IL-6 following multi-species probiotics have all reported mean reductions in average IL-6 levels from baseline. Three of these presented significant changes from baseline; Sabico et al. (123) reported a difference of –3.9 pg/mL (-76.5%), while Kobyliak et al. (136, 138) reported changes of 3.45 ± 1.48 and –6.77 ± 9.62 pg/mL across two studies. Another study by Kobyliak et al. (137) revealed statistically significant changes of –4.11 ± 7.15 pg/mL from baseline compared to control. Other studies have also reported decreasing averages in IL-6 levels post supplementation, however, these changes were not found to be statistically significant (128, 129).

##### Effect on IFN-γ

Two studies by Kobliyak et al. (136, 137) have investigated and reported the effect of multi-species probiotics on IFN-γ; no studies have investigated the effects of single species probiotic supplementation on this pro-inflammatory marker (Table 2). These two “Symbiter Forte” regimens were associated with net decreases in IFN- γ, although statistically non-significant.

##### Effect on IL-8

Three multispecies probiotic administering probiotic combinations have been associated with significant efficacies following an 8-week intervention (Table 2). Kobliyak et al. (136–138) have reported reductions in IL-8 levels ranging from –3.80 ± 1.05 to –8.99 ± 21.11 pg/mL, although it must be noted that all three interventions were not statistically significant from the effect seen in control.

##### Effect on Interleukin 17

Only Hsieh et al. (134) have reported on the association of IL-17 levels with probiotic administration (Table 2). However, the authors report statistically insignificant effects; changes of + 0.66 ± 3.24 and + 0.47 ± 2.91 ng/mL following live and heat-killed probiotic use.

##### Effect on Endotoxin

Two studies by Sabico et al. (123, 140) have investigated the effects of multi-species probiotics on blood endotoxin levels in diabetic patients (Table 2). While one study reported that following probiotic supplementation, endotoxin levels significantly dropped by 3.2 IU/mL (-69.6%) from baseline, another reported a significant change of –2.40 IU/mL (-52.2%) compared to the change seen in control.

##### Effect on Lipopolysaccharide-Binding Protein

The one study reporting on the effect of probiotic on LBP concluded that there was no change in the mean levels of marker following a 16-week intervention with *Lacticaseibacillus casei* (135).

#### Anti-inflammatory Markers in Type 2 Diabetes

##### Effect on Interleukin-10

A study by Hsieh et al. (134) investigated the effect of a 6-month single species probiotic course on diabetic patients; although there was a generally positive trend in the change post-intervention compared to baseline and control (+ 1.48 ± 3.09 and + 2.05 ± 3.25 ng/mL in the live and heat-killed bacteria groups, respectively), these results were not statistically significant.

##### Effect on IL-1RA

Andreasen et al. (132) have reported an insignificant increase of + 2 pg/mL in IL-1RA from baseline following a 4-week regimen of single-species probiotic; however, this change was much more pronounced in control.

#### Markers of Oxidative Stress in Type 2 Diabetes

##### Effect on Malondialdehyde

Malondialdehyde (MDA) is one of the final products of polyunsaturated fatty acids peroxidation in the cells. An increase in free radicals causes overproduction of MDA. MDA has been extensively studied as a biomarker of inflammation in the context of metabolic disease. A total of seven studies investigated this inflammatory marker as a subject of change following supplementation with either single (2) or multispecies (5) probiotics (Table 2).

Miraghajani et al. (141) reported a beneficial 0.07 μmol/L change in mean MDA levels among a cohort of T2D patients with nephropathy after an 8-week course of single-species probiotics that was significantly different than the change observed among controls receiving conventional soy milk. On the other hand, another group reported an non-significant increase of 0.06 μmol/L following a course of another single-species probiotic in bread (142).

Among five multi-species probiotics investigated for their effects on MDA levels among diabetics, two have reported significant reductions in MDA levels, while three have presented statistically insignificant results. Ejtahed et al. (143) have reported a significant decrease of 0.26 μmol/L in the mean level of MDA from baseline following a 6-week course, while a more recent study by Raygan et al. (127) have presented a significant change of –0.1 ± 0.3 μmol/L from baseline compared to control following a twice-longer course. Mazloom et al. (128) and Raygan et al. (126) both reported net reductions in mean level of MDA markers; however, these were not found to be statistically significant.

##### Effect on Superoxide Dismutase

SOD levels post probiotic administration has been a subject of investigation under four studies included in this review (Table 2). All studies have reported positive trends, some have been reported as significant versus control. Mirmiranpour et al. (144) have reported slightly higher SOD levels post-intervention than control after the authors report that no significant change existed between the groups at baseline. Hariri et al. (145) have also presented the significant effects of another single-species probiotic on diabetic nephropathy patients following an 8-week course. In another study, the same authors reported changes of + 0.93 ± 0.95 and + 0.04 ± 1.31 U/mL after administration of live and heat-killed bacterium, however, these were not found to be statistically significant (134). Lastly, Ejtahed et al. (143) reported a significant increase of 137.87 U/g Hb in erythrocyte SOD from baseline following a 6-week course of a multi-species probiotic yogurt.

##### Effect on Catalase

A few studies have reported on the effect of nutraceuticals on CAT levels (Table 2). While one study reported a significant result, an overall conclusion cannot be made in this regard as two others have found no statistically significant changes nor similar trends. Mirmiranpour et al. (144) have reported that after starting from similar baseline values, the group receiving 3 months of a single-species probiotic capsule (2.44 ± 0.50 U/mL) had markedly increased CAT levels compared to control (1.95 ± 0.34 U/mL), a difference in mean of 0.49 U/mL that was statistically significant. While a slight positive change from baseline was also found by Bahmani et al. (142) this was not significantly different from the change observed in control; on the other hand, Ejtahed et al. (143) found an overall insignificant decrease in mean CAT.

##### Effect on GSH Peroxidase and GSH Reductase

Multiple studies have investigated the effect of probiotics on GPX activity levels post-intervention, however, the results have been conflicting (Table 2). Ejtahed et al. (143) reported that a 6-week regimen of multi-species probiotic was associated with a significant increase in mean GPX activity to hemoglobin ratio of 0.78 U/g Hb compared to both baseline and control. Similar significant increases (of + 0.43 U/g Hb in GPX and + 0.38 U/g Hb in GR) have been reported by Miraghajani et al. (141) following an 8-week administration of a single-species probiotic. More recently, Mirmiranpour et al. (144) have reported that after starting from similar levels at baseline, the difference between an intervention group receiving single-species probiotic and control was + 7.26 U/mL in GPX activity. Interestingly, another group of researchers have shown fractional and statistically insignificant changes in GPX activity after single-species probiotic activity; however, these changes were smaller in magnitude than that seen in control (134).

##### Effect on GSH and GSSG

The effect of probiotics on reduced (GSH) and oxidized (GSSG) glutathione levels have been studied across multiple studies with interstudy consenting trends (Table 2). All studies report a positive change in mean plasma GSH following intervention periods, although not all have been found to be statistically significant. Miraghajani et al. (141) reported a statistically significant increase from baseline in GSH (732.96 ± 61.95 vs. 600.66 ± 69.61 μmol/L) and a similar decrease in GSSG (19.00 ± 0.70 vs. 30.37 ± 0.20 μmol/L) following an 8-week supplementation period with a single-species probiotic soymilk; these changes were also statistically significant compared to changes observed in a control group. Although Bahmani et al. (142) also investigated another single-species probiotic in this regard, the results were not statistically significant given a larger increase in the control group.

Among multispecies probiotics investigation, Asemi et al. (124) reported a significant increase of + 240.63 ± 101.29 μmol/L from baseline compared to control the earliest. Raygan et al. (125–127) through a series of three, 3-months studies investigating different multi-species probiotics, a change of + 18.0 ± 112.7 μmol/L from baseline in one of the studies was reported, and baseline-adjusted intergroup mean difference of changes of + 45.15 and + 154.16 μmol/L was reported in the two other studies; all three reports were statistically significant.

##### Effect on Total Antioxidant Capacity and Total Antioxidant Status

The association of probiotic supplementation on the levels of TAC has been extensively studied (Table 2). While many of these reports a significant increase in mean levels of TAC, few others have presented a mean positive change that are not statistically significant.

Miraghajani et al. (141) and Bahmani et al. (142) have reported respective changes of + 39 and + 78.6 mmol/L in mean TAC levels among diabetics following an 8-week supplementation with different single-species probiotics; however, these were not statistically significant. Among the other studies reporting on the effect of multispecies probiotics, only Asemi et al. (124) failed to show a statistically significant change despite a large mean increase of + 379.97 ± 41.8 mmol/L from baseline levels. On the other hand, Raygan et al. (127) have shown that a mean increase of just 12.6 ± 41.6 mmol/L was significant due to the control group having a large decrease of –116.9 ± 324.2 mmol/L. However, through a series of two other multispecies probiotic-investigating studies among diabetics with congestive heart disease, Raygan et al. (125, 126) have reported larger significant baseline-adjusted intergroup mean differences of + 108.44 and + 119.30 mmol/L. Ejtahed et al. (143) and Tonucci et al. (146) have also presented significant decreases in TAS levels compared to baseline and control group, despite a less pronounced change.

##### Effect on Nitric Oxide

Few studies have investigated changes in NO levels as a measure of oxidative stress following probiotic use (Table 2). Bahmani et al. (142) reported a statistically non-significant rise (+ 18.5 ± 36.2 μmol/L) in mean NO levels following 8 weeks of single-species probiotic bread supplementation. On the other hand, Raygan et al. (125–127) successfully reported, through three different studies each using multispecies probiotics, net increases in mean NO levels in the order of + 7.86 μmol/L and + 4.28 μmol/L (both as intragroup baseline adjusted intergroup mean difference) and + 1.7 ± 4.0 μmol/L (change from baseline).

##### Effect on Oxidized Low-Density Lipoprotein, 8-Hydroxy-2’-Deoxyguanosine and F_2_-Isoprostanes

OxLDL, 8-OhdG and F_2_-IsoP remain some of the lesser used measures of oxidative stress utilized to investigate oxidative stress (Table 2). Mirmiranpour et al. (144) recently reported that compared to a control group (17.07 ± 1.01 mU/L) that did not have significantly different mean OxLDL levels at baseline, an intervention of single-species probiotics was associated with a lower mean OxLDL (16.85 ± 1.53 mU/L) at 3 months; however, this was not statistically significant. Similarly, Tonucci et al. (146) reported a decrease in F_2_-IsoP levels following a course of multispecies probiotics. Hariri et al. (145) described a significant intergroup mean decrease in 8-OhdG, however specific marker levels were not reported.

#### Pro-inflammatory Markers in Type 2 Diabetes

##### Effect on C-Reactive Protein and High-Sensitivity C-Reactive Protein

We found six studies investigating the effects of prebiotics on hs-CRP, with most studies reporting significant (*p* ≤ 0.05) decreases in this marker of inflammation following administration of inulin, resistant dextrin, or resistant starch in T2D patients (Table 3). Dehghan et al. have reported that high performance (HP) inulin supplementation in T2D patients resulted in a significant baseline adjusted mean hs-CRP difference of –3.8 ng/mL compared to a control group consuming maltodextrin, while another recent study has confirmed this significant effect of HP inulin using a cohort of diabetic and overweight patients consuming either HP inulin or HP inulin plus butyrate (147, 148). Moreover, a recent study by Farhangi et al. (149) reported that resistant dextrin supplementation was responsible for a significant baseline-adjusted mean difference of –8.02 ng/mL (-54.00%) compared to a similar control. Resistant dextrin has also been showed to be associated with a significant reduction of –2.40 ng/mL in hs-CRP from baseline in one study by Aliasgharzadeh et al. (47) Administration of oligofructose-enriched inulin and a formula containing 60% resistant starch type 2 were also shown to result in promising baseline-adjusted mean differences of –3.9 ng/mL (-31.70%) and –4.6 ng/mL (-11.9%) compared to their control(s), respectively, although these reductions were not found to be statistically significant (150, 151).

**TABLE 3:** Studies investigating the effect of prebiotics on markers of inflammation and oxidative stress in T2D.

Type of nutraceutical	Study Design, Country	Participant* demographics size/sex (n, F/M) age (Mean ± SD; yrs.) BMI (Mean ± SD; kg/m^2^)		Control/Placebo substance administered	Interventional nutraceutical administered	Control/Placebo and intervention dose × frequency	Total Period of intervention/ study	Effect on markers	Mean change in Φ	References
		Control/Placebo	Intervention							
Prebiotic	TB, PC, RCT Iran	*n* = 25 (25F) 49.6 ± 8.4 30.8 ± 5.2	*n* = 30 (30F) 49.2 ± 9.6 31.8 ± 4.5	Maltodextrin	Resistant dextrin	5 x 2 g/d	8 weeks	↓IL-6	−1.45 pg/ml or −28.4%	(47)
								↓TNF-α	−5.40 pg/ml or −18.8%	
								↓MDA	−1.21 nmol/ml or −25.6%	
								↓Endotoxin	−6.2 units/ml or −17.8%	
								↓hs-CRP (§)	−2.7 mg/L or 35.1 % (§)	
								↓hs-CRP	−2.40 mg/L	
Prebiotic	R, TB, PC (Iran)	*n* = 25 (25F) 48.40 ± 9.70 29.90 ± 4.10	*n* = 27 (27F) 48.45 ± 8.40 31.90 ± 4.00	Maltodextrin	Oligofructose-enriched inulin	5 x 2 g/d	8 weeks	↑TAC	+0.2 mmol/L or +20.0%	(155)
								↑CAT (§)	69.5 ± 20.2 U/mg Hb (I, 8w) vs 57.2 ± 16.0 U/mg Hb ↑(I, B)	
								↑GPX (§)	34.4 ± 5.4 U/mg Hb (I, 8w) vs 33.7 ± 5.1 U/mg Hb (I, B) (§)	
								↑SOD (§)	1684.7 ± 254.2 U/mg Hb (I, 8w) vs 1633.9 ± 237.3 U/mg Hb (I, B)	
								↓MDA	−1.7 nmol/mL or −39.7%	
Prebiotic	TB, RCT (Iran)	*n* = 25 (25F) 48.7 ± 9.7 29.9 ± 4.2	*n* = 27 (27F) 48.4 ± 8.4 31.9 ± 4.5	Maltodextrin	Oligofructose-enriched Inulin	5 × 2 g/d	8 weeks	↓hs-CRP (§)	−3.9 mg/L or −31.70% (§)	(147)
								↓TNF-α	−3.0 pg/ml or −19.80%	
								↓IL-6	−1.3 pg/ml or −8.15%	
								↓LPS	−6.0 EU/mL or −21.95%	
								↓IFN-λ (§)	−0.3 pg/ml or −16.50% (§)	
								↑IL-10 (§)	+0.4 pg/ml or +11.50% (§)	
Prebiotic	R, DB, PC, CT (Iran)	T2D and overweight patients* n = 15 (5M/10F) 51.73 ± 8.44 30.86 ± 5.41	T2D and overweight patients* Inulin group *n* = 15 (8M/7F) 51.47 ± 6.46 30.37 ± 2.82	Starch powder and starch capsules	HP inulin, starch capsules as placebo	(C) 6 x100 mg/d starch capsules, 5 x 2 g/d starch powder (I) 2 × 5 g/d HP inulin, 6 × 100 mg starch capsules/d	45 days	↓hs-CRP	3.80 ± 1.38 mg/L (I, 45d) vs 5.45 ± 2.28 mg/L (I, B) [−25.63%];	
									significant MD vs control, markers NS	(149)
								↓MDA	6.13 ± 1.93 nmol/mL (I, 45d) vs 6.40 ± 2.09 nmol/mL (I, B) [−3.39%];	
									significant MD vs control, markers NS	
								↓TNF-α mRNA	Ratio: 0.75 ± 0.18-fold vs baseline;	
									also significant vs control, markers NS	
			T2D and overweight patients*; Butyrate + Inulin group *n* = 14 (4M/10F) 47.14 ± 7.99 30.31 ± 4.25	Starch powder and starch capsules	NaBut, HP Inulin	(C) 6 x100 mg/d starch capsules, 5 x 2 g/d starch powder (I) 2 × 5 g/d HP inulin, 6 × 100 mg NaBut capsules	45 days	↓hs-CRP	2.44 ± 1.01 mg/L (I, 45d) vs 3.89 ± 1.14 (I, B) [−34.25%];	
									significant MD vs control, markers NS	
								↓MDA	5.51 ± 2.17 nmol/mL (I, 45d) vs 6.68 ± 2.27 nmol/mL (I, B) [−12.31%];	
									significant MD vs control, markers NS	
								↓TNF-α mRNA	Ratio: 0.91 ± 0.32 fold vs baseline;	
									also significant vs control, markers NS	
Prebiotic	R, DB, C, CT (Iran)	Control Group *n* = 27 (12M/15F) 58.2 ± 11.8 BMI NR	Prebiotic Group *n* = 28 (14M/16F) 58.8 ± 12.8 BMI NR	Capsule containing 0.5 g of rice flour powder	Capsule containing 0.5 g of powdered cinnamon	1 capsule/d	3 months	↑CAT	2.44 ± 0.50 U/ml (I, 3m) vs 1.95 ± 0.34 U/ml (C, 3m)	(144)
								^.^GPX (§)	84.61 ± 13.43 U/mL (I, 3m) vs 84.89 ± 6.52 U/mL (C, 3m) (§)	
								↑SOD (§)	4.16 ± 0.60 U/mL (I, 3m) vs 3.99 ± 0.27 U/mL (C, 3m) (§)	
								↓OxLDL (§)	16.32 ± 1.21 mU/L (I, 3m) vs 17.07 ± 1.01 mU/L (C, 3m) (§)	
Prebiotic	TB, RCT (Iran)	*n* = 32 (32F) 49.6 ± 8.4 30.8 ± 5.2	*n* = 28 (28F) 49.5 ± 8.0 31.5 ± 4.5	Maltodextrin	Hi-Maize 260 (60% resistant starch type 2)	5 x 2 g/d	8 weeks	↓hs-CRP (§)	−4.6 mg/L or −11.9% (§)	(152)
								↓IL-6 (§)	−1.4 pg/ml or −14.2% (§)	
								↓TNF-α	−3.4 pg/ml or −18.9%	
Prebiotic	DB PC (Iran)	T2D and overweight patients* n = 22 (22F) 48.61 ± 9.16 29.98 ± 4.01	T2D and overweight patients* n = 27 (27F) 48.07 ± 8.70 31.43 ± 3.50	Maltodextrin	Oligofructose-enriched chicory inulin	5 x 2 g/d	2 months	↑IL-4	7.41 ± 1.38 pg/ml vs −2.96 ± 0.88 pg/ml	(153)
								↓IL-12	−2.49 ± 1.60 pg/ml vs +1.23 ± 0.60 pg/ml	
								↓IFN-λ	−0.28 ± 0.06 pg/ml vs +0.058 ± 0.03 pg/ml	
Prebiotic	R, PC, CT (Iran)	*n* = 25 (25F) 48.7 ± 9.7 29.9 ± 4.2	*n* = 24 (24F) 47.8 ± 10.1 31.6 ± 4.1	Maltodextrin	HP inulin	5 x 2 g/d	8 weeks	↓hs-CRP	−3.8 mg/L	(148)
								↓TNF-α	−2.9 pg/ml	
								↑IL-10	+1.9 pg/ml	
								↓LPS	−4.2 EU/mL	
Prebiotic	TB, PC, RCT (Iran)	*n* = 25 (25F) 49.6 ± 8.4 30.8 ± 5.2	*n* = 30 (30F) 49.2 ± 9.6 31.8 ± 4.5	Maltodextrin	Resistant Dextrin	5 x 2 g/d	8 weeks	↓IL-12	−2.8 pg/ml	(154)
								↓IL-12 (§)	−0.7 pg/ml (§)	
								↑IL-4 (§)	+4.3 pg/ml	
								↓IL-4 (§)	−1.0 pg/ml (§)	
								↑IL-10	+2.6 pg/ml	
								↓IFN-λ	−0.6 pg/ml	
								↓IFN-λ/IL-10 ratio	−0.01	
								↓LPS	−6.1 EU/mL	
Prebiotic	R, DB, PC (S. Korea)	IFG, IGT and T2D patients* n = 25 (10M/15F) 56.0 ± 1.28 24.6 ± 0.50	IFG, IGT and T2D patients* n = 22 (4M/18F) 54.4 ± 1.31 23.8 ± 0.63	Powdered rice flour	Jerusalem artichoke (containing inulin and fructooligosaccharides) and fermented soybean powder mixture 1:1	40 g/d	12 weeks	↓MDA (§)	−0.41 ± 0.43 nmol/L vs −0.58 ± 0.35 nmol/L (§)	(156)
Prebiotic	R, DB, PC (Japan)	*n* = 25 (17M/8F) 54 ± 12 27.2 ± 4.6	*n* = 27 (21M/6F) 55 ± 11 27.9 ± 3.6	Maltodextrin syrup	Galacto-oligosaccharide syrup	10 g/d	4 weeks	IL-6 (§)	2.3 ± 4.3 pg/ml (I, 4w) vs 2.3 ± 4.8 pg/ml (I, B) (§)	(38)
								↓ IL-10 (§)	3.3 ± 7.7 pg/ml (I, 4w) vs 3.3 ± 7.7 pg/ml (I, B) (§)	
								↓ TNF-α (§)	2.2 ± 1.4 pg/ml (I, 4w) vs 2.5 ± 2.3 pg/ml (I, B) (§)	
								↓ LBP (§)	12.6 ± 2.2 μg/mL (I, 4w) vs 12.8 ± 1.8 μg/mL (I, B) (§)	
Prebiotic	R, PC, CT (Iran)	*n* = 33 (33F) 48.6 ± 7.9 32.0 ± 3.9	*n* = 32 (32F) 49.5 ± 8.0 31.5 ± 4.5	Maltodextrin	Resistant dextrin supplement (NUTRIOSE06)	5 x 2 g/d	8 weeks	↑TAC	+0.33 mmol/L or +36.25%	(150)
								↓hs-CRP	−8.02 mg/L or −54.00%	
								↓LPS	−6.5 EU/mL or −23.40%	
								↑SOD	+56.3 U/mg Hb	
								↑SOD (§)	+67.5 U/mg Hb (§)	
								↑GPX	+3.80 U/g Hb	
								↑GPX (§)	+0.85 U/g Hb (§)	
								↓CAT (§)	−5.3 U/g Hb	
								↓CAT (§)	−10.7 U/g Hb (§)	
								↓MDA	−1.21 nmol/mL or −25.58%	

*All participants are T2D-diagnosed patients, unless otherwise stated; ↓ indicates a decrease in value; ↑ indicates an increase in value; ^.^ = No change in value; Φ = Order of markers compared = those of Intervention (I) group first, Control (C) or baseline (B) second; Text color represents comparison body as follows, Blue = Comparison of effective change due to intervention by adjusted Mean Difference (MD) of changes in markers observed between I&C groups at end of the study from baseline; Green = Comparison of changes in I vs. C groups at the end of study from respective baselines; Red = Comparison of change (or difference) in markers at the end of study from baseline in I group; § = Non-significant Result; T2D = Type-2 Diabetes; NS = Not Specified; NR = Not Reported; SB = Single-Blinded; DB = Double-Blinded; TB = Triple-Blinded; R = Randomized; RCT = Randomized Controlled Trial; CC = Crossover Controlled; PC = Placebo-Controlled; PG = Parallel Group; CT = Clinical Trial;; OL = Open Label; MDA = Malondialdehyde; IL-6 = Interleukin 6; TNF-α = Tumor necrosis factor alpha; CRP = C-reactive protein; SCFA = Short-Chain Fatty Acid; NAFLD = Non-Alcoholic Fatty Liver Disease; GSH: Glutathione; GSSG = oxidized glutathione; Hs-CRP: High sensitivity C-Reactive Protein; CAT = Catalase; TAC = Total Antioxidant Capacity; TAS = Total Antioxidant Status; LPS = lipopolysaccharide; LBP = lipopolysaccharide binding protein; IL-1ra = IL-1 receptor antagonist; SOD = Superoxide Dismutase; GPX = Glutathione Peroxidase; GR = glutathione reductase; HP = High Performance; F2-iso = F2-isoprostane; IFG = impaired fasting glucose; IGT = impaired glucose tolerance; NGT = normal glucose tolerance.

##### Effect on Tumor Necrosis Factor-α

Five out of six studies investigating the effect of prebiotic supplementation on TNF-α have reported promising results, while another has reported a non-significant (*p* > 0.05) decrease in the levels of this inflammatory marker (Table 3) (38). Aliasgharzadeh et al. (47) have reported a significant baseline-adjusted mean difference of 5.40 pg/mL (-18.8%) between the prebiotic group receiving HP inulin and control. Two other studies have reported similar baseline-adjusted differences of 3.0 pg/mL (-19.80%) and 2.9 pg/mL between changes of prebiotic and control groups following supplementation with oligofructose-enriched and HP-inulin, respectively (147, 150). Similar results have been reported from the use of resistant starch by Gargari et al. while significant reductions of 25 and 9% in baseline have been reported following administration of inulin with starch or butyrate, respectively, by another group (148, 151).

##### Effect on Interleukin-6

Prebiotic supplementation on the levels of IL-6 has been investigated in four studies, two of which have reported significant decrease in levels compared to control after adjusting for baseline values, while two other studies investigating resistant starch and galacto-oligosaccharides have found statistically insignificant results (Table 3) (38, 151). Aliasgharzadeh et al. have reported an adjusted change of –1.45 pg/mL (-28.4%) in IL-6 compared to control following an 8-week course of resistant dextrin, while Dehghan et al. have reported a –1.3 pg/mL (-8.15%) in baseline-adjusted changes in IL-6 vs. control following an 8-week course of oligofructose-enriched Inulin (47, 150).

##### Effect on Interleukin-12

Two studies have reported the effect of prebiotics on the pro-inflammatory cytokine IL-12 with promising success (Table 3). Dehghan et al. reported that a 10 g/d regimen with prebiotics was associated with a significant reduction of 2.49 ± 1.60 pg/mL in IL-12 levels compared to the change of + 1.23 ± 0.60 pg/mL in those receiving placebo (152). Farhangi et al. also reported a significant change of –2.8 pg/mL from baseline in another group of diabetics receiving a different prebiotic; however, comparison with control after adjusting for baseline values did not yield significant results (153).

##### Effect on IFN-γ

Unlike the inconclusive results of the multispecies probiotic studies discussed before, of three studies investigating the effect of prebiotics on IFN-γ, two reported favorable results that were statistically significant. Only an early study by Dehghan et al. reported statistically insignificant mean difference of –0.3 pg/mL (16.50%) between intergroup effects (150). Later, Dehghan et al. and Farhangi et al. reported a change of –0.28 ± 0.06 pg/mL from baseline and a baseline-adjusted mean difference of –0.6 pg/mL between intervention and control, respectively (152, 153). The latter group also noted a significant reduction in the IFN- γ/IL-10 ratio of –0.01 (153).

##### Effect on Endotoxin or Lipopolysaccharide

A total of five studies have investigated the effects of prebiotics on blood endotoxin levels in diabetic patients, a cross-study trends point to prebiotics having a significant negative effect overall (Table 3). Aliasgharzadeh et al. have reported a significant baseline-adjusted mean difference of –6.2 units/mL following intervention with resistant dextrin (-17.8%) (47). Similar significant effects of resistant dextrin administration have been published by two other studies: –6.5 EU/mL (-23.40%), (149) –6.1 EU/mL (153). HP and oligofructose-enriched inulin were also shown to also be significantly negatively associated LPS levels: –4.2 and –6.0 EU/mL (-21.95%), respectively, (147, 150).

##### Effect on Lipopolysaccharide-Binding Protein

Gonai et al. are the only group that have reported the results of prebiotics on LBP; no mean statistically significant change was observed following a 4-week intervention with galacto-oligosaccharides (Table 3) (38).

#### Adaptive Immunity Markers in Type 2 Diabetes

##### Effect on IL-4

More research is required in context to the effect of pre- and synbiotics, on IL-4 post-intervention (Table 3). Dehghan et al. have reported that HP inulin significantly increased IL-4 levels by + 7.41 ± 1.38 pg/mL compared to control in a cohort of diabetic and overweight patients (152). However, more recent study by Farhangi et al. produced inconclusive results as the change due to intervention was positive compared to baseline, but negative compared to the baseline-adjusted change in control; both of these were statistically insignificant (153).

#### Anti-inflammatory Markers in Type 2 Diabetes

##### Effect on IL-10

Four studies have reported the effects of prebiotic supplementation in diabetic patients, with the majority following a trend of positive change compared to baseline and/or control (Table 3). Dehghan et al. reported a significant baseline-adjusted mean difference of + 1.9 pg/mL between changes in IL-10 post 8 weeks of intervention with HP inulin compared to control (147). Another study has also reported a similar significant mean difference of + 2.6 pg/mL using resistant dextrin (153). In addition, a study by Dehghan et al. also reported a positive change in IL-10 levels, however, this was not found to be statistically significant, similar to the results of Gonai et al. (38, 150).

#### Markers of Oxidative Stress in Type 2 Diabetes

##### Effect on Malondialdehyde

MDA levels have also been widely probed following prebiotic use; four studies have investigated and reported significant baseline-adjusted intergroup mean difference between intragroup changes, while another has reported changes from baseline (Table 3). Aliasgharzadeh et al. have reported net effects of intervention of –1.21 nmol/mL (-25.6%) and –1.7 nmol/mL (-39.7%) using two different prebiotics (47, 154). Farhangi et al. have recently confirmed the effects of resistant dextrin in another study, reporting an intervention effect of –1.21 nmol/mL (-25.58%) (149). Finally, Roshanravan et al. have interestingly also reported the effect of HP inulin with and without butyrate; both effects were significant with mean changes of 1.17 and 0.27 nmol/mL from baseline levels, respectively, after just 45 days (148). Finally, Ahn et al. reported a slight decrease in MDA levels from baseline using Jerusalem artichoke and soyabean mixture, but this was not found to be significant (155).

##### Effect on Superoxide Dismutase

Like probiotics, all three studies considered exploring the effect of prebiotics on SOD levels have reported positive changes (Table 3). Farhangi et al. have recently reported a significant increase of + 56.3 U/mg Hb from baseline in SOD levels after an 8-week course with resistant dextrin (149). Aliasgharzadeh et al. and Mirmiranpour et al. have also investigated the effects of other prebiotics on SOD levels; however, these were statistically insignificant (144, 154).

##### Effect on Catalase

Three trials administering prebiotics have reported on their effects on CAT levels, with little evidence to support any one hypothesis (Table 3). Mirmiranpour et al. have reported that a 3 months course of prebiotics was significantly associated with increased CAT activity levels (+ 2.44 ± 0.50 U/mL) compared to the control group (1.95 ± 0.34 U/mL) when baseline levels were not significantly different (144). Furthermore, a similar increase in mean CAT/Hb ratio (+ 12.3 U/g Hb) in the intervention group was reported by another group, this, however, was not statistically significant (154). Only Farhangi et al. reported a non-significant decrease in mean CAT following prebiotic use (149).

##### Effect on GPX

Three distinct clinical studies have reported the use of prebiotics to investigate changes in GPX levels among diabetic patients (Table 3). Of these, only Farhangi et al. have presented results that were significant; following an 8-week course of prebiotics, a difference of + 3.80 U/g Hb was observed from baseline in the intervention group (149). Aliasgharzadeh et al. and Mirmiranpour et al. have also reported on GPX levels post-prebiotic administration; however, almost no change was observed in both cases following supplementation using their respective prebiotics (144, 154).

##### Effect on Total Antioxidant Capacity

Two studies have investigated the effect of prebiotics on TAC levels, with each reporting a significant increase post-supplementation (Table 3). Aliasgharzadeh et al. reported a mean difference of + 0.2 mmol/L (+ 20.0%) associated with the prebiotic intervention (154), while Farhangi et al. more recently have reported a larger change of + 0.33 mmol/L (36.25%) (149).

##### Effect on OxLDL

Mirmiranpour et al. reported that compared to a control group without significantly different baseline OxLDL levels, an intervention of prebiotic was associated with a lower mean OxLDL (16.32 ± 1.21 vs. 17.07 ± 1.01 mU/L) at 3 months; however, this was not statistically significant (Table 3) (144).

### Effect of Synbiotics on Inflammatory and Oxidative Stress Markers in Type 2 Diabetes

#### Pro-inflammatory Markers in Type 2 Diabetes

##### Effect on CRP and hs-CRP

A total of eight studies reported the association between synbiotic consumption in diabetics and either CRP or hs-CRP, with many studies reporting significant desired results (Table 4). Moreover, like the trend observed in probiotics, multispecies synbiotic supplementation outperform single-species probiotics in their ability to result in desired changes in CRP and hs-CRP levels.

**TABLE 4:** Studies investigating the effects of synbiotics on markers of inflammation and oxidative stress.

Type of nutra-ceutical	Study Design, Country	Participant* demographics size/sex (n, F/M) age (Mean ± SD; yrs.) BMI (Mean ± SD; kg/m^2^)		Control/Placebo substance administered	Interventional nutraceutical administered		Control/placebo and intervention dose x frequency	Total period of intervention/ study	Effect on change in		Mean change in markers Φ		References
		Control/Placebo	Intervention										
Synbiotic (Single sp.)	R, DB, CC, CT (Iran)	*n* = 62 (19M/43F) 53.1 ± 8.7 29.90 ± 5.18	*n* = 62 (19M/43F) 53.1 ± 8.7 29.60 ± 4.53	0.38 g isomalt, 0.36 g sorbitol and 0.05 g stevia per 1g		Heat-resistant Bacillus coagulans (1 × 10^7^ CFU), 0.04 g inulin (HPX), 0.38 g isomalt, 0.36 g sorbitol and 0.05 g stevia per 1g		9 × 3 g/d	6 × 2 weeks	↓hs-CRP		−1.057.86 ± 283.74 ng/ml vs +95.40 ± 385.38 ng/ml	(157)
										↑GSH		+319.98 μmol/L vs. +19.73 μmol/L	
										↑TAC (§)		+69.48 ± 38.13 mmol/l vs +60.06 ± 40.76 mmol/l (§)	
Synbiotic (Single sp.)	DB, R, CC, CT (Iran)	*n* = 51 (16M/35F) 52.9 ± 8.1 30.15 ± 5.07	*n* = 51 (16M/35F) 52.9 ± 8.1 29.88 ± 4.77	0.38 g isomalt, 0.36 g sorbitol and 0.05 g stevia per 1 g		*Bacillus coagulans* (1 × 10^7^ CFU), 0.1 g inulin HPX), 0.05 g beta-carotene with 0.38 g isomalt, 0.36 g sorbitol and 0.05 g stevia per 1 g		9 x 3 g/d	6 x 2 weeks	↑NO		+6.83 ± 16.14 μmol/L vs. −3.76 ± 16.47 μmol/L	(159)
										↑GSH		+36.58 ± 296.71 μmol/L vs. −92.04 ± 243.05 μmol/L	
										↓TAC (§)		−6.97 ± 203.51 mmol/L vs. −10.03 ± 170.15 mmol/L	
										↓hs-CRP (§)		−274.70 ± 3560.67 ng/mL vs −212.02 ± 2943.09 ng/mL (§)	
										↓MDA		−1.28 ± 1.33 μmol/l	
										↓MDA (§)		−1.28 ± 1.33 μmol/l vs −0.95 ± 0.88 μmol/l (§)	
Synbiotic (Single sp.)	R, DB, C, CT (Iran)	Control Bread (CB) *n* = 25 (Sex NS) 54.60 ± 0.83 27.04 ± 0.50 Lactic Acid Bread (LAB) *n* = 25 (Sex NS) 55.00 ± 0.97 26.33 ± 0.46	Synbiotic group *n* = 25 (Sex NS) 54.92 ± 1.02 26.39 ± 0.51	Bread containing beta- glucan (3g) ± lactic acid (4 g)/ 40g package		Bread containing beta-glucan (3 g), *Bacillus coagulans* (1 × 10^8^ CFU), and inulin (10 g)/40g package		40g x 3 packages/d	8 weeks	↓TAC (§)		−0.007 ± 0.01 mmol/L vs – 0.01 ± 0.01 mmol/L (CB) & +0.02 ± 0.02 mmol/L (LAB) (§)	(158)
										↑SOD		+0.40 ± 0.13 mmol/L vs +0.18 ± 0.17 mmol/L (CB) & −0.54 ± 0.40 mmol/L (LAB)	
										↑GPX (§)		+0.85 μmol/L vs +0.47 U/mL (CB) & +1.23 U/mL (LAB) (§)	
										↓hs-CRP		−689.76 ± 368.98 ng/mL vs +33.80 ± 237.60 ng/mL (LAB)	
			Synbiotic + Lactic Acid group *n* = 25 (Sex NS) 53.88 ± 1.09 26.83 ± 0.42	Bread containing beta- glucan (3g) ± lactic acid (4 g)/40g package		Bread containing beta-glucan (3 g), *Bacillus coagulans* (1 × 10^8^ CFU), inulin (10 g) and lactic acid (4 g)/40g package		40g x 3 packages/d	8 weeks	↑TAC (§)		+0.03 ± 0.01 mmol/L vs – 0.01 ± 0.01 mmol/L (CB) & +0.02 ± 0.02 mmol/L (LAB) (§)	
										↑SOD		+0.87 ± 0.22 mmol/L vs +0.18 ± 0.17 mmol/L (CB) & −0.54 ± 0.40 mmol/L (LAB)	
										↑GPX		+19.02 ± 17.10 U/mL vs. −24.05 ± 12.17 U/mL (LAB)	
										↓hs-CRP (§)		−575.96 ± 268.60 ng/mL vs +519.35 ± 304.35 ng/mL (CB) & +33.80 ± 237.60 ng/mL (LAB) (§)	
Synbiotic (Single sp.)	R, DB, C, CT (Iran)	Control Bread (CB) *n* = 27 (5M/22F) 53.4 ± 7.5 30.5 ± 4.1	Synbiotic Bread *n* = 27 (5M/22F) 51.3 ± 10.4 30.8 ± 5.9	Control bread		Bread containing viable and heat-resistant *Bacillus coagulans* (1 × 10^8^ CFU) and 0.07g inulin/1g		40 x 3 g/d	8 weeks	↓hs-CRP (§)		–983.5 ± 2,066.1 ng/ml vs –586.9 ± 2,009.2 ng/ml (§)	(160)
Synbiotic (Single sp.)	R, DB, C, CT (Iran)	Control Group *n* = 27 (12M/15F) 58.2 ± 11.8 BMI NR	Synbiotic Group *n* = 30 (Sex NS) 58.4 ± 11.4 30.8 ± 5.9 BMI NR	Capsule containing 0.5 g of rice flour powder		Capsule containing *Lactobacillus acidophilus* (10^8^ CFU) and 0.5 g of powdered cinnamon		1 capsule/d	3 months	↑CAT (§)		2.20 ± 0.31 U/ml (I, 3m) vs 1.95 ± 0.34 U/ml (C, 3m) (§)	(144)
										↑GPX (§)		89.71 ± 9.04 U/mL (I, 3m) vs 84.89 ± 6.52 U/mL (C, 3m) (§)	
										↑SOD (§)		4.13 ± 0.64 U/mL (I, 3m) vs 3.99 ± 0.27 U/mL (C, 3m) (§)	
										↓OxLDL		15.88 ± 1.98 mU/L (I, 3m) vs 17.07 ± 1.01 mU/L (C, 3m)	
Synbiotic (Single Sp.)	R, DB, C, CT (Iran)	Control Bread (CB) *n* = 27 (5M/22F) 53.4 ± 7.5 30.5 ± 4.1	Synbiotic Bread *n* = 27 (5M/22F) 51.3 ± 10.4 30.8 ± 5.9	Control bread		Bread containing viable and heat-resistant *Bacillus coagulans* (1 × 10^8^ CFU) and 0.07g inulin/1g		40 x 3 g/d	8 weeks	↑NO		+40.6 ± 34.4 μmol/L vs −0.8 ± 24.5 μmol/L	(142)
										↑TAC (§)		+3.6 ± 247.2 mmol/L vs. -45.7 ± 240.3 mmol/L (§)	
										↑GSH (§)		+25.0 ± 528.2 μmol/L vs +18.8 ± 417.8 μmol/L	
										↑CAT (§)		+2.2 ± 25.7 U/mL vs +2.7 ± 14.9 U/mL	
Synbiotic (Multi Sp.)	R, DB, PC, CT (Iran)	Overweight and obese patients with T2D and CHD* n = 30 (11M/19F) 64.0 ± 11.7 29.6 ± 4.6	Overweight and obese patients with T2D and CHD* n = 30 (11M/19F) 64.2 ± 12.0 32.3 ± 6.0	Capsules containing placebo		Capsules containing *Lactobacillus acidophilus* T16, L. casei T2 and Bifidobacterium bifidum strain T1 (2 × 10^9^ CFU/g each) and 800 mg inulin		1 capsule/d	12 weeks	↓hs-CRP		−2632.3 ± 743.2 ng/ml vs – 433.3 ± 743.2 ng/ml	(162)
										↑NO		+7.6 ± 1.7 μmol/L vs – 3.4 ± 1.7 μmol/L	
										↑TAC (§)		+49.8 ± 33.6 mmol/L vs. +30.0 ± 33.6 mmol/L (§)	
										↑GSH (§)		+23.6 ± 17.1 μmmol/l vs +12.2 ± 17.1 μmmol/l (§)	
										↓MDA		−0.6 ± 0.1 μmmol/l vs – 0.1 ± 0.1 μmmol/l	
Synbiotic (Multi Sp.)	R, DB, PC (Iran)	T2D and non-obese patients* n = 23 (14M/9F) 60.39 ± 6.74↑↑ 28.27 ± 2.54	T2D and non-obese patients* n = 20 (12M/8F) ↑59.10 ± ↑9.71 27.32 ± 4.34	Sachet containing 2g starch and 0.7% Natural Orange flavor		2g sachet containing 10^11^ spores of *Bacillus coagulans* Ganeden BC30, 10^10^ CFU *Lacticaseibacillus rhamnosus* GG,↑10^9^ CFU *Lactobacillus acidophilus*, 500↑mg fructooligosaccharides and 0.7% Natural orange flavor		1 x 2 g/d	12 weeks	↓hs-CRP		−2.41 ± 2.48 mg/L↑vs +0.89 ± 3.21 mg/L	(163)
Synbiotic (Multi Sp.)	RCT, OL (Japan)	T2D and obese patients* n = 42 (34M/8F) 55.9 ± 10.7 29.1 ± 3.	T2D and obese patients* n = 44 (31M/13F) 61.1 ± 11.0 29.5 ± 4.4	NS, no pre- pro- or synbiotics		Dry powder (dp) containing *Lacticaseibacillus paracasei* YIT 9029 (3 × 10^8^ CFU), *Bifidobacterium breve* YIT 12272 (3 × 10^8^ CFU), and 7.5g galacto-oligosaccharides (GOS)		(2gdp, 5gGOS) + (1gdp, 2.5gGOS)/d	24 weeks	↓IL-6 (§)		−0.2 ± 1.8 pg/ml vs +0.4 ± 2.0 pg/ml	(161)
										↑CRP (§)		+40.0 mg/dL vs -3.5 mg/dL (§)	
										↑LBP (§)		+2.0 ± 4.2 μg/mL vs 3.4 ± 4.2 μg/mL (§)	
Synbiotic (Multi Sp.)	R, PG, DB, PC (Iran)	Patients with T2D and chronic periodontitis* n = 24 (8M/16F) 50.1 ± 3.6 25.5 ± 2.7	Patients with T2D and chronic periodontitis* n = 23 (6M/17F) 48.6 ± 5.8 24 ± 3.6	Same substance as intervention without bacteria and fructo-oligosaccharide		500 mg capsule containing 7 viable and freeze-dried strains: *Lactobacillus acidophilus* UBLA-34 (2 × 10^9^ CFU), L. casei (7 × 10^9^ CFU), *L. rhamnosus* (1.5 × 10^9^ CFU), *L. bulgaricus* (2 × 10^8^ CFU), *Bifidobacterium breve* (2 × 10^9^ CFU), *B. longum* (7 × 10^9^ CFU), *Streptococcus thermophilus* (1.5 × 10^9^ CFU) and 100 mg fructo-oligosaccharide		1 capsule/d	8 weeks	↓IL-1β		−0.45 ± 0.42 pg/ml vs −0.13 ± 0.47 pg/ml	(165)
										↓MDA		−1.02 ± 0.95 μM vs −0.13 ± 0.39 μM	
										↑TAC		+0.04 ± 0.06 mM	
										↑TAC (§)		+0.04 ± 0.06 mM vs +0.01 ± 0.12 mM (§)	
										↑SOD		+1.75 ± 2.49 U/mL vs +0.16 ± 0.48 U/mL	
										↑CAT (§)		+0.44 ± 5.33 U/mL vs +0.44 ± 5.33 U/mL	
										↑GPX		+14.72 ± 24.9 U/mL vs −3.27 ± 22.86 U/mL	
Synbiotic (Multi sp.)	R, DB, PC, Pilot (Austria)	Diabesity patients* n = 14 (8M/6F) 59 34	Diabesity patients* n = 12 (11M/1F) 61 33	Probiotic matrix containing maize starch, maltodex-trins, vegetable protein, potassium chloride, magnesium sulphate, amylases and manganese sulphate and prebiotic matrix containing maltodextrin, natural elderflower flavoring and Gum Arabic		Probiotic Ecologic Barrier containing g B. bifidum W23, *Bifidobacterium animalis* subsp. lactisW51, *Bifidobacterium animalis* subsp. lactisW52, *Lactobacillus acidophilus* W37, *L. casei* W56, *L. brevis* W63, *L. salivarius* W24, *L. lactis* W58 and *L. lactis* W19 (1.5 × 10^10^ CFU total) and 6 g matrix and 10 g, and Prebiotic ‘Omnilogic Plus’ containing 8 g active Galacto-oligosaccharides and Fructo-oligosaccharides, konjac glucomannan, calcium carbonate, zinc citrate 3-hydrate, vitamin D3 (cholecalciferol) and vitamin B2 (riboflavin) and 2g matrix		1 each/d	6 months	↑LPS (§)		0.69 EU/mL (I, 6m) vs 0.64 EU/mL (mg/dL) (§)	(166)
										↑LBP (§)		20.5 ng/mL (I, 6m) vs. 19 ng/mL (I, B) (§)	
Synbiotic (Sp. NS)	R, DB, PC (Iran)	*n* = 22 (8M/14F) 54.5 ± 11.10 22.47 ± 2.38	*n* = 22 (8M/14F) 53.45 ± 10.8 22.79 ± 2.7	Placebo tablet		Synbiotic tablet		1 tablet/d	8 weeks	↓hs-CRP		4.15 ± 1.96 mg/L (I, 8w) vs 4.94 ± 2.36 mg/L (I, B);	
												significant MD vs control, markers NS	(164)
										↓IL-6		8.12 ± 5.02 ng/L (I, 8w) vs 9.19 ± 5.97 ng/L (I, B);	
												significant MD vs control, markers NS	
										↓TNF-α		7.36 ± 2.61 ng/L (I, 8w) vs 8.03 ± 2.73 ng/L (I, B);	
												significant MD vs control, markers NS	

*All participants are T2D-diagnosed patients, unless otherwise stated; ↓ indicates a decrease in value; ↑ indicates an increase in value; ∙ = No change in value; Φ = Order of markers compared = those of Intervention (I) group first, Control (C) or baseline (B) second; Text color represents comparison body as follows, Blue = Comparison of effective change due to intervention by adjusted Mean Difference (MD) of changes in markers observed between I&C groups at end of the study from baseline; Green = Comparison of changes in I vs. C groups at the end of study from respective baselines; Red = Comparison of change (or difference) in markers at the end of study from baseline in I group; § = Non-significant Result; T2D = Type-2 Diabetes; NS = Not Specified; NR = Not Reported; Sp. = Species; SB = Single-Blinded; DB = Double-Blinded; TB = Triple-Blinded; R = Randomized; RCT = Randomized Controlled Trial; CC = Crossover Controlled; PC = Placebo-Controlled; PG = Parallel Group; CT = Clinical Trial; OL = Open Label; MDA = Malondialdehyde; IL-6 = Interleukin 6; TNF-α = Tumor necrosis factor alpha; CRP = C-reactive protein; SCFA = Short-Chain Fatty Acid; NAFLD = Non-Alcoholic Fatty Liver Disease; GSH: Glutathione; GSSG = oxidized glutathione; Hs-CRP: High sensitivity C-Reactive Protein; CAT = Catalase; TAC = Total Antioxidant Capacity; TAS = Total Antioxidant Status; LPS = lipopolysaccharide; LBP = lipopolysaccharide binding protein; IL-1ra = IL-1 receptor antagonist SOD = Superoxide Dismutase; GPX = Glutathione Peroxidase; GR = glutathione reductase; HP = High Performance; F2-iso = F2-isoprostane; IFG = impaired fasting glucose; IGT = impaired glucose tolerance; NGT = normal glucose tolerance.

Asemi et al. reported that a single species synbiotic mixture of *Bacillus coagulans* (previously *Lactobacillus sporogenes*) and inulin significantly correlated to hs-CRP level changes from baseline of –1.057 ± 0.283 mg/L compared to a slight mean increase of 0.0054 ± 0.385 mg/L in control, whereas another intervention group from a study using *B. coagulans* and inulin rich bread presented a significant change of –689.76 ± 368.98 μg/L compared to an increase of 33.80 ± 237.60 μg/L in a control group consuming lactic acid-enriched bread (156, 157). Similar studies investigating the effects of single species synbiotics using other formulations of *B. coagulans* and inulin have all showed a trend of decrease in the levels of either CRP or hs-CRP, although these results were not significant (157–159).

Among multi-species synbiotic nutraceuticals, only Kanazawa et al. recently reported a non-significant increase of 40.0 mg/dL in CRP from baseline following supplementation with *Lacticaseibacillus paracasei, Bifidobacterium breve* and galacto-oligosaccharides (GOS) in a cohort of diabetic and obese patients in Japan (160). However, two other studies based in Iran investigating cohorts with similar comorbidities reported significant inter-group reductions in the levels of hs-CRP compared to controls following supplementation consisting of *Lactobacillus acidophilus, L. casei, Bifidobacterium bifidum* and inulin, and *B. Coagulans, Lacticaseibacillus rhamnosus, Lactobacillus acidophilus* and fructooligosaccharides, respectively, (161, 162). A study by Kooshki et al. (163) reported significant decrease in hs-CRP from both baseline vs. control, although the composition of the tablet was not specified.

##### Effect on TNF-α

It is interesting to note that the effect of synbiotics on TNF-α is minimally reported (Table 4). Kooshki et al. (163) have found that multi-species synbiotic supplementation led to a mean decrease of ∼0.67 pg/mL following administration of a synbiotic tablet; however, the composition of this supplement was not reported.

##### Effect on IL-1β

Like TNF-α, the data on synbiotic supplementation on IL-1β is currently limited and requires further research (Table 4). Nonetheless, Bazyar et al. (164) have reported significant changes of –0.45 ± 0.42 pg/mL compared to the effect of control following an 8-week supplementation with a 7-species plus fructo-oligosaccharides synbiotic in a cohort of diabetics with chronic periodontitis from Iran.

##### Effect on IL-6

There is a limitation in the literature exploring the effects of synbiotic supplementation on IL-6, with only two studies reporting an overall average decrease in IL-6. However, only one of those reports was found to be statistically significant (Table 4). Kooshki et al. have reported a significant ∼1 ng/L change from baseline in IL-6 compared to control following an 8-week supplementation of synbiotic (composition unreported) (163). A further statistically insignificant change of –0.2 ± 1.8 pg/mL in IL-6 was reported by another group following a 24-week regimen of galacto-oligosaccharide and multispecies synbiotic (160).

##### Effect on Endotoxin (LPS)

Endotoxin (LPS) levels have not been widely investigated in the context of synbiotic administration (Table 4). Nevertheless, it is interesting to note that Horvath et al. have recently described a mean increase of 0.05 EU/mL from a baseline of 0.64 EU/mL in LPS levels following a 6-month intervention with a multi-species probiotic; however, this was not found to be significant with respect to intragroup baseline or the intergroup (control), perhaps owing to the small sample size in the pilot study (165).

##### Effect on Lipopolysaccharide-Binding Protein

Multispecies synbiotic intervention was associated with a larger change in mean LBP levels than either pro- or synbiotics, although both studies investigating their association failed to show statistically significant results (Table 4). Kanazawa et al. and Horvath et al. reported an increases in average marker levels from baseline (+ 2.0 ± 4.2 μg/mL and + 1.5 ng/mL, respectively) following a 6-month intervention (160, 165).

#### Markers of Oxidative Stress in Type 2 Diabetes

##### Effect on Malondialdehyde

MDA levels have been investigated as a marker of oxidative stress following synbiotic use in three studies; all three have reported some form of significant decrease in mean levels of MDA following supplementation (Table 4). Asemi et al. reported that a single-species probiotic was associated with a significant decrease of –1.28 ± 1.33 μmol/L from baseline; however, this was not significant compared to the change seen in control (158). However, two recent studies by Farrokhian et al. and Bazyar et al. have yielded significant changes of –0.6 ± 0.1 and –1.02 ± 0.95 μmmol/L following multi-species probiotic use (161, 164).

##### Effect on Superoxide Dismutase

The effect of synbiotics on SOD levels in diabetics were investigated by three studies, all of whom have reported an increasing effect following intervention (Table 4). Ghafouri et al. reported significant rise in SOD levels of + 0.40 ± 0.13 and + 0.87 ± 0.22 mmol/L following an 8-week supplementation with bread containing single-species probiotics without and with lactic acid, respectively (157). Mirmiranpour et al. presented a statistically insignificant increase of 0.14 U/mL in mean SOD levels between the two groups (144). While Bazyar et al. reported a large increase of + 1.75 ± 2.49 U/mL following a similar time frame of intervention using a multi-species probiotic in diabetic patients with chronic periodontitis (164).

##### Effect on Catalase

While three trials have reported the effect of various synbiotics on CAT activities, these were not found to be statistically significant, despite all showing upward slopes following intervention (Table 4). Mirmiranpour et al. reported that the mean CAT activities between the group receiving single-species synbiotic and the control differed by 0.25 U/mL at the end of trial, while another study reported a change of + 2.2 ± 25.7 U/mL from baseline in the intervention group (142, 144). Finally, although Bazyar et al. reported a slight increase in CAT activity from baseline, the change was found not to differ from control (164).

##### Effect on GPX

The results of synbiotic use on diabetics with respect to GPX has been investigated in three studies showing promising results (Table 4). Bazyar et al. has recently presented a significant promising change of + 14.72 ± 24.9 U/mL from baseline compared to control (164). On the other hand, Ghafouri et al. has reported another significant change of + 19.02 ± 17.10 U/mL in GPX following administration of a bread containing another synbiotic combination (157). The results of Mirmiranpour et al. show promise in their large mean increase from baseline compared to control, however, it was not statistically significant (144).

##### Effect on GSH

The effect of synbiotics on mean reduced glutathione (GSH) levels have also been investigated across literature; while all of five such studies report an increase in the mean GSH, only two have shown to be statistically significant (Table 4). Through two similar single-species synbiotics, Asemi et al. have shown increases of + 319.98 (vs. + 19.73 μmol/L in control) and + 36.58 ± 296.71 (vs. –92.04 ± 243.05 μmol/L in control) (156, 158). A more recent study by Raygan et al. demonstrated an increase in mean GSH (+ 18.0 ± 112.7 vs. –12.2 ± 122.5 μmol/L in control) following another single-species synbiotic supplement, these effects, however, were not statistically significant (127). In another study investigating multi-species synbiotic use among diabetics failed to show significant results given a similar rise in mean GSH in control (142). In addition, a recent study among severely comorbid diabetics had also failed to reach statistical significance in the findings (161).

##### Effect on Total Antioxidant Capacity

While multiple studies have investigated the effect of synbiotic use on TAC, the overall trends are not conclusive, with only a single study reporting a statistically significant association (Table 4). Bazyar et al. have reported a significant increase in TAC of + 0.04 ± 0.06 mM from baseline following a multispecies probiotic supplementation; however, this was not significant when compared to the change in control (164). On the other hand, Farrokhian et al. reported a larger absolute increase in mean TAC level (+ 49.8 ± 33.6 mmol/L), however, it was also not significant compared to a the change of + 30.0 ± 33.6 mmol/L in control (161).

Among single-species probiotics, Asemi et al. reported, across two studies, mean changes of + 69.48 ± 38.13 and –6.97 ± 203.51 mmol/L following different formulations; however, in both instances, the average change was positive when compared to control, although statistically insignificant (156, 158). A similar (yet statistically insignificant) trend is seen in the case of Bahmani et al. wherein a change of + 3.6 ± 247.2 mmol/L was found among the intervention group compared to –45.7 ± 240.3 mmol/L among the controls (142). Finally, Ghafouri et al. compared the effects of synbiotic bread with or without a metabolite, however, the effect of both on TAC levels were found to be both statistically and numerically insignificant (157).

##### Effect on Nitric Oxide

Very few studies have reported on the effect of NO following synbiotic supplementation among diabetics; however, of the available studies, all have shown significant associations following synbiotic supplementation (Table 4). Bahmani et al. reported an increase of + 40.6 ± 34.4 μmol/L in NO levels following an 8-week course of single-species synbiotic bread (142), while Asemi et al. reported an increase of + 6.83 ± 16.14 μmol/L following a 6 × 2 weeks crossover trial of another singles-species synbiotic (158). On the other hand, Farrokhian et al. showed a significant increase of + 7.6 ± 1.7 μmol/L after a 12 week intervention with a multi-species synbiotic (161).

##### Effect on Oxidized Low-Density Lipoprotein

Mirmiranpour et al. reported that compared to a baseline matched control group, an intervention of single-species synbiotic was significantly associated with a lower mean OxLDL (15.88 ± 1.98 vs. 17.07 ± 1.01 mU/L) at 3 months (Table 4) (144).

## Discussion

This systematic review collectively pooled data from forty-seven randomized controlled trial (RCT) studies to investigate the effect of probiotic, prebiotic and synbiotic supplementation on various markers of inflammation and oxidative stress among patients with T2D, with or without other comorbidities. Our results point toward the successful capacity of gut-microbiome promoting therapeutics to have beneficial effects on multimodal inflammation and oxidative stress inducing factors in the pathogenesis of T2D. Here we discuss intervention-specific trends in our findings that may add to growing evidence of currently researched questions and/or incentivize novel discoveries.

### The Promise of Probiotics

Described as “live microbial feed supplements which beneficially affect the host animal by improving its intestinal microbial balance,” (166) probiotics have been widely studied across literature given their capacity to have clinical therapeutic potential by antagonizing pathogenic or “harmful” bacteria and/or reversing pathogenic dysbiosis of the microbiome (166–170). Despite their role in metabolic diseases only being described from 2007 after a description of their potential role in obesogenesis in mice (171), a large array of mechanisms listing their potential against T2D and clinical trials testing their efficacies have surfaced.

A recent systematic review of association of different bacterial gut microbiome species in the T2D pathophysiology has reported that most species of *Bifidobacterium* are associated with a protective function in T2D, with lower levels of this probiotic compared to healthy controls being reported in T2D patients (172). Strain and species-specific associations of (previously) *Lactobacillus* have also been reported, although with more complexity in interpretation, given apparent opposite effects of different strains. Among studies investigating the effect of probiotics on inflammatory and oxidative stress biomarkers, multi-species probiotic supplementation has shown to be largely consistently more effective compared to single-species or monostrain probiotic administration. This can be observed in pro-inflammatory markers like hs-CRP (and CRP), TNF-α, IL-β, IL-6, IL-8, and LPS (endotoxin) across multiple trials (123, 136–140). Among these, (previously) *Lactobacillus* and *Bifidobacterium* strains such as *L. casei, L. rhamnosus, L. gasseri, L. plantarum, B. infantis, B. longum*, and *B. breve*, often in synergism, and to a lesser extent, *Lactococcus, Propionibacterium*, and *Acetobacter* strains, have been reported to be consistently promising. *Bifidobacterium* have been shown across multiple animal studies to have promising probiotic effects in multiple metabolic dysfunctions through a variety of mechanisms; these include restoring the lymphocyte-macrophage balance and gut microbiota structure, reducing B-cell infiltration and increasing Treg activity (173–175), and modulating gut microbiome resulting in higher acetate SCFA levels (176). Animal models have also cemented that *Bifidobacterium* and (previously) *Lactobacillus* strains including *B. adolescentis* N3, *B. adolescentis* 7-2, *B. bifidum* M2, *L. rhamnosus* 7-1, and *L. rhamnosus* YC, were independently correlated with reduced levels of inflammatory biomarkers such as TNFA, IL1B, and IL-6 (177). However, given that (previously) *Lactobacillus* single-species administration has been associated with insignificant differences across multiple clinical trials reported in this review (124, 129, 143, 178), we may conclude that their probiotic effects are best attained in humans when used in combination with other strains and species of the same or different genus. This superiority of multistrain and multispecies probiotic is not new and has been described across other diseases and trials of the gut, such as pouchitis and ulcerative colitis (179). This is likely due to the enhanced probability of at least one of the many strains/species administered to survive, adapt, and produce anti-pathogenic and dysbiosis-attenuating response upon administration and survival in diseased microbiome consisting of harmful bacteria, whereas a single species is more vulnerable to endogenous microflora. In addition, single-species probiotics are limited in their therapeutic potential given that they are limited to the species-specific ability of the probiotic to render beneficial changes, whereas a collection of multispecies bacteria may not only have multiple mechanisms of completing similar beneficial pathways but may also use different pathways to achieve similar end effects.

Additionally, probiotics like *Lactobacillus acidophilus*, *L. reuteri, L. fermentum, L. bulgaricus, Bifidobacterium bifidum*, *Bifidobacterium animalis* subsp. *Lactis*, and *Streptococcus thermophilus* have shown significant promise with respect to markers such as MDA (127), SOD, (143), GPX (144), GSH (124), TAC, and NO (125). Possible mechanisms reported include their effects on ascorbate autoxidation, metal-ions chelation, antioxidant enzymes system, and various antioxidant metabolites such as GSH (180), butyrate, and folate (181), and activity reduction and excretion of free radicals such as superoxide anion and hydrogen peroxide (182, 183). Their mode of delivery includes fermented milk, yogurt, bread, or simply as supplemental capsules. Hence a combination with sources that are shown to have probiotic-independent antioxidant abilities such as that of casein-derived peptides should be considered to maximize effectiveness (184). Using animal models, Hsieh et al. have shown that multi-strain probiotic composed of *Lactobaccilus salivarius* subsp. *salicinius* AP-32, *L. johnsonii* MH-68, *L. reuteri* GL-104, and *Bifidobacterium animalis* subsp. *lactis* CP-9 improved not only inflammatory markers, but also reduced MDA and increased SOD levels (185), potentially having β-cell protective function which would otherwise be disrupted due to oxidative stress (186).

### The Promise of Prebiotics

Prebiotics refer to non-digestible and fermentable food ingredients serving as substrates that are selectively utilized by host gut-microbiota to provide health benefits through encouraging the growth of beneficial bacteria (187, 188). They are abundantly found in multiple fruits, vegetables, and cereals, including bananas, beans, garlic, onions, peas, and artichoke in the form of polysaccharides such as inulin, oligosaccharides, including both galactooligosaccharides and fructooligosaccharides, resistant starch, and even cinnamon (144, 167). By traveling undigested through the upper GI system, they are available for fermentation by the beneficial bacteria in the colon (189), leading to the production of beneficial metabolites such as SCFAs (acetate, butyrate, propionate) and lactic acid, which has significant effects on inflammation and intestinal membrane integrity (26), along with other mechanisms.

In our review, although we have highlighted the promise offered by a variety of prebiotic supplementations, resistant dextrin has been shown to be the most widely effective in both categories of markers (147, 149, 152, 153). Resistant dextrins are non-sweet short-chain glucose polymers with high resistance to digestive enzymes of the human gut, up to 75% of which is available for fermentation (172, 190). Ślizewska et al. have shown that resistant dextrin supplementation among mice was associated with lower levels of *Clostridium* strain (191), which is found in elevated levels among diabetics compared to normal gut (192). Resistant dextrin also led to higher levels of both the beneficial *Bifidobacterium* and prior *Lactobacillus* strains in the faces and cecum of rats without changing the overall bacterial count significantly (191). Similar results have been reported from clinical trials involving dextrin supplementation in humans (193). Valcheva et al. reported that IL-10 deficient mice fed with fiber dextrin diets over the course of 12 weeks secreted 47–88% less colonic IL-1β, TNF-α, IL-23, IL-12p70, IL-6, and CXCL1, with lower enterocyte injury scores and an increase in butyrate SCFA production (194). It has been priorly described (150) that through production of NF-κβ, butyrate modulates inflammation, controls macrophage and neutrophil activators and chemoattractant (195), and increases the expression of cytokine signaling 3 suppressor (196), all of which serve to promote anti-inflammatory Th2-lymphocyte differentiation rather than into Th1, ultimately increasing IL-10 levels among other mechanisms (197).

The prebiotic is also associated with beneficial changes in serum insulin, lipid, and gut microbial composition, with the promotion of the insulin signaling and the fatty acid β oxidation pathways in high-fat-high-fructose diet-fed rats and the enhancement of *Parasutterella* and *Parabacteroid* relative abundances and prevention of further harmful gut dysbiosis (198). Total SCFA concentrations and those of acetate, butyrate, and propionate individually were found to be dose-dependently higher among a group of rats fed increasing amounts of resistant dextrin compared to control (199). Finally, among two placebo-controlled RCTs investigating NUTRIOSE, a commercial resistant dextrin formula, increased levels of *Bacteroides* and SCFAs were observed (199). Resistant dextrin has also shown to significantly increase GSH/GSSG ratio (by 33%) in rat models (200). By promoting growth of acid-resistant bacteria (such as *Roseburia*) that produce butyrate, resistant dextrin has shown to reduce ROS levels and increase plasma antioxidant enzymes through an LPS-mediated process that includes multiple factors such as NF-κβ (201–203). In addition, inhibiting the growth of *Clostridium perfringens* has been shown to reduce ROS through PKC, MEK/ERK, and NFκB pathways (149). Other mechanisms through which prebiotics including resistant dextrin but also oligo-fructose-enriched inulin, may modulate oxidative stress markers include reducing advanced glycation end -products and by serving as scavengers of ROS (150). Our findings are consistent with prior reviews (188) in this field.

### The Promise of Synbiotics

Synbiotics are combinations of probiotic and prebiotic that are administered together. The rationale behind acceptance of their co-administration has been multifaceted: it has been shown to improve probiotic survival through provision of metabolic substrates that facilitate gastrointestinal tract transition, increase viability (204) and possible synergistic effects that may be independent of but parallel to the effects of the probiotic itself (205). Such combinations have shown to have beneficial effects on insulin resistance and glucose metabolism (25). Moreover, adding a prebiotic such as inulin to probiotic milk, yogurt, ice cream, and cheese formulations was found to increase survival in storage, increase apparent viscosity (206). Green et al. have also argued that the rationale of specific matching of prebiotic and probiotic can also include the possibility that certain prebiotics promote the growth of some bacteria more than others, if at all, based on the findings of Scott et al. who report that chain length of fructans (in prebiotic) is an important factor determining fermentation-specificity between species (207). Perhaps the most significant effect of a synbiotic is conferred from the study by Nazzaro et al. where the authors show that *Lactobacillus acidophilus* growth using inulin was associated with 14.5 times more butyrate production than in the presence of pectin, both of which were still significantly higher than administration of the probiotic with just glucose, with undetectable levels of butyrate (208). In addition to the priorly discussed roles of SCFAs such as butyrate, it has also been shown that butyrate inhibits IFN-γ production and has an active role in regulating peroxisome proliferator activated receptor-g (209). Clinical trials have reported more effective beneficial effect of synbiotic administration than in the case of probiotics alone in the case of prediabetic individuals with focus on prediabetes (210).

In our study, we have shown that across multiple studies investigating probiotics, prebiotics and synbiotics, the latter group of supplements have shown to be more effective compared to the former two primarily when comparing the change in oxidative stress biomarkers and that of antioxidant enzymes, namely MDA (164), plasma (164) and erythrocyte SOD (143), GPX (157), GSH (156), NO (142), and OxLDL (144). A combination of multispecies probiotic of *Lactobacillus acidophilus, L. casei, L. rhamnosus, L. bulgaricus, Bifidobacterium breve, B. longum, Streptococcus thermophilus*, and the prebiotic fructo-oligosaccharide has shown exemplary promise among the greatest number of markers. *Bacillus coagulans* with inulin was the most effective single species synbiotic. Given the previously described rising importance to “mix-and-match” prebiotic with a probiotic that can derive maximal benefit from it, the work of Fuhren et al. with respect to synbiotic matchmaking using inulin, fructooligosaccharides and multiple strains of *Lactiplantibacillus plantarum* show an effective screening model that may enable researchers and industrial manufacturers to create the ideal synbiotic (211). One such study by Nagpal and Kaur revealed that *L. casei*, one of the seven probiotic species of the most promising synbiotic identified in this review, had higher viability in inulin compared to oligosaccharide media (212); it would be interesting to compare the other strains and species with various prebiotics, especially resistant dextrin, which was identified as the most promising in this review.

### Intestinal Dysbiosis and Inflammation

Apart from the direct impact of poor nutrition on the immune response, changes in the intestinal microbiota due to obesogenic environmental factors can also stimulate inflammation (213). The altered composition of the gut microbiota due to the consumption of a high-fat, low-fiber diet has been directly correlated with a pronounced low-grade inflammatory state linked to T2D (214). This is because consumed fibers are broken down and fermented by the gut microbiota in the large intestine into short chain fatty acids (SCFAs), including butyrate, propionate, and acetate (215). These metabolites are recognized by GPR41 and GPR43, both of which are G protein-coupled receptor. SCFAs are considered a main source of nutrients and energy for colonocytes and microbes, are involved in the regulation of energy and pH levels, and stimulate the release of glucagon-like peptide-1 (GLP-1) (216). Lower fiber intake results in a decrease in metabolites such as SCFAs and eventually lead to cases of intestinal dysbiosis. Microbial metabolites also have significant implications on inflammatory responses. Butyrate controls the maturation of dendritic cells (DC) as well as preventing associations between adipocytes and macrophages (217). Propionate, on the other hand, decreases synthesis of adipokine in adipocytes (218). Acetate also participates in the maintenance of balance in the gut by suppressing the expression of inflammatory cytokines while upregulating the synthesis of anti-inflammatory cytokines (26). Thus, a decrease in these metabolites due to reduced fiber intake can stimulate pro-inflammatory responses in patients with T2D. Additionally, dysbiosis allows potentially pathogenic microbes to shift locations and replicate faster in the gut epithelium. Large numbers of translocated bacteria are recognized as invaders by the immune system (219), thereby evoking a chronic inflammatory response through the activation of toll-like receptors (TLRs) and upregulation of pro-inflammatory cytokines (220).

Since there is a proven alteration in the gut microbiota of T2D, researchers have investigated the merit of correcting this dysbiosis as a potential cure for T2D. It is unclear if measurable alteration in the gut microbiota directly correlates to or is needed for pro/prebiotics to exert their effect for treating T2D (165). The short study duration of some of the clinical studies can explain this; for example, Gonai et al. (38) have shown that while the prebiotic galacto-oligosaccharide (GOS) ameliorated the decrease of *Bifidobacteriaceae* in T2D, LPS- binding protein (LBP) and glucose tolerance did not improve during the trial period. LPB stimulates inflammatory cytokines through TLR4 and thus it provides a potential mechanism of how the gut microbiota dysbiosis causes T2D (38). However, in another small study examining the effect of GOS on gut permeability no significant improvement has been found (221). It is important to note that the first study had a bigger sample size and a larger administered dose of GOS than the second. Another prebiotic fiber that is extensively studied in the treatment of T2D is inulin-type fructans. Increased production of SCFAs improves T2D and a 50/50 mixture of inulin and oligofructose increases *bifidobacteria* and SCFAs in feces, while butyric acid and the microbial diversity are not affected (41).

### Intestinal Permeability and Inflammation

The intestinal barrier is a semipermeable membrane that regulates the absorption of nutrients and electrolytes from the lumen into the blood stream, while preventing the entrance of infectious microorganisms as well as antigens, endotoxins, and proinflammatory substances (222). Intestinal permeability, therefore, is an intrinsic characteristic of the intestinal epithelium that allows for the exchange of luminal substances whilst maintaining an immunological barrier. However, intestinal hyperpermeability, commonly referred to as a “leaky gut,” has been linked to several disorders, including gastrointestinal, such as celiac disease and colon carcinoma, in addition to other extra-intestinal diseases, including diabetes (223). Even though defective intestinal barriers could be a result of disease aggravation, clinical studies hypothesize that it could also be a causal factor in the progression of disease and the initiation of autoimmune destruction (224). Altered intestinal barrier function leads to an unrestricted influx of antigens or toxins into the gut, consequently instigating an inflammatory response in the lumen and other proximal organs (225). Increased intestinal permeability has, thus, become a new target for disease prevention and therapy of type 1 and 2 diabetes (226). Considering the close relationship between intestinal permeability and gut dysbiosis, we can conclude that meticulous dietetic and probiotic approaches to recover healthy microbiota have the potential to make a breakthrough in the management of these diseases in the near future.

An epidemiological study has shown that the increased levels of 16S ribosomal DNA from gut bacteria in the blood is a risk factor for diabetes (227). Thus, it is hypothesized that bacterial translocation could play a role in T2D pathophysiology. It has been found that the administration of the probiotic *Lacticaseibacillus casei* strain Shirota could reduce gut bacterial translocation and can alter the gut microbiota in patients with T2D (135). Additionally, *L. reuteri* and *L. gasseri* both were found in higher levels in the probiotic fed subjects’ fecal samples. This may explain the decreased bacterial translocation as these bacteria improve the membrane integrity through mucus production expression of tight junctions and reduction of apoptosis (135). Further, it was also shown by Horvath et al. (165) that multispecies synbiotic strengthens the gut barrier function and thus reduces levels of c-peptide, LPS and bacterial DNA in the participants’ serum, indicating a decreased translocation of bacterial products to the blood.

### Gut Microbiota, Nutraceuticals, COVID-19, and Diabetes

While age remains to be the most significant predictor of COVID-19 related morbidity and mortality, diabetes, along with other chronic conditions, was identified early as a significant comorbidity of the disease (228), with diabetes being present in over one-third of hospitalized individuals in one New York City cohort (11). Low-grade inflammation, characterized by chronically increased levels of inflammatory cytokines such as IL-6, TNF-α, and IL-1β, is present as a common feature of many metabolic diseases including T2D, as well as COVID-19 (229). Since diabetics with low-grade inflammation compared to healthy controls have shown notable reductions in serum inflammatory markers following insulin therapy (230) and COVID-19 associated mortality among diabetics was also significant reduced in a subgroup with controlled blood glucose (231), it may very well be the case that control of low-grade inflammation through modulation and correction of gut dysbiosis may serve as a significant therapeutic strategy against COVID-19 associated mortality and morbidity among those with pre-existing T2D. Perhaps unsurprisingly, COVID-19 hospitalized patients have been shown to have “significant alterations in fecal microbiomes, characterized by enrichment of opportunistic pathogens (such as *Coprobacillus, Clostridium ramosum*, and *Clostridium hathewayi*) and depletion of beneficial commensals (such as *Faecalibacterium prausnitzii*, an anti-inflammatory bacterium, and multiple *Bacteroides* species responsible for downregulation of ACE2 expression)” (232). It is then expected that probiotics may be able to assist host innate and adaptive immunity in COVID-19 struck patients as a form of adjuvant strategy (233). In addition to resolution of gut dysbiosis, probiotic have the potential to contribute to a healthy gut-lung axis by reducing translocation of pathogens through the intestinal mucosa, thereby reducing the potential of simultaneous infections, which may lead to poorer prognosis. The effects of bacteria from the previously *Lactobacillus* and *Bifidobacterium* genus have been elucidated in our systematic review; it is very encouraging to also note that these species have shown great promise in reducing the incidence of Ventilator associated pneumonia and upper-respiratory tract infections in venerable cohorts (234). In fact, probiotics have been shown to have a larger beneficial effect in inflammatory biomarker levels such as IL-6 and CRP as a measure of low-grade inflammation than even Angiotensin Receptors Blockers, omega-3, metformin, resveratrol, and vitamin D (235). These gut-microbiome modulating nutraceuticals have shown to serve also as immunomodulators leading to downregulation of the low-grade inflammation state (236, 237), leading to an overall reduction or attenuation of COVID-19 related symptoms such as “diarrhea, abdominal pain, vomiting, headache, cough, sore throat, fever, and viral infection complications such as acute respiratory distress syndrome (ARDS)” (238). By close monitoring of potential interactions with diabetes and antiviral drugs, especially conserving that many antibiotics given during COVID-19 infection may further lead to gut microbiome dysbiosis (233), probiotics, along with pre- and synbiotics, may serve as therapeutic agents with low adverse event incidence for treatment of diabetics with COVID-19 (239).

## Conclusion

**FIGURE 2:**
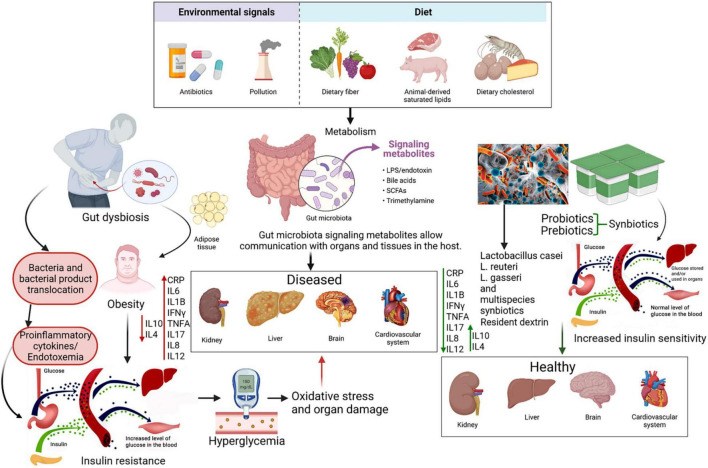
Summary diagram illustrating the effect of diet, pre and probiotics as a potential safe therapy in treating T2D.

Systematic review of current literature showed that T2D patients have a different gut microbiome composition than healthy individuals which may result from dysbiosis due to the pathogenic state. On the other hand, it is also plausible that this altered gut microbiota has the potential to lead early onset and development of T2D. By altering the gut microbiota using pre-, pro-, and synbiotics, it is possible to modify factors causing inflammation and oxidative stress. In this review, we have reported on multiple promising and effective pre-, pro-, and synbiotics for their association to changes observed in markers of inflammation and oxidative stress. We have identified significant trends and observations between single and multistrain probiotics, identified the most promising rising synbiotic as resistant dextrin, and showed how synbiotics may be more effective compared to the other two types of nutraceuticals among oxidative stress markers. Furthermore, we have elucidated and reviewed the role of metabolites, signaling pathways, low-grade inflammation, gut permeability, and dysbiosis with respect their ultimate roles in the pathogenesis and possible therapy in T2D, as well as the potential of these nutraceuticals to attenuate COVID-19 infection related symptoms. Figure 2 provides a generalized summary of the role of microbiome-targeted nutraceuticals in T2D pathogenesis and potential correction of dysbiosis.

## Final Remarks, Prospects and Clinical Trials

While human clinical trials in literature have elucidated the great potential and promise of using pro-, pre-, and synbiotics as therapeutic agents for the treatment of T2D, further large-scale and multicenter trials and investigations are required given their controversial results in the past. What also needs to be assessed and delivered is the type of biological agents that are most likely to be accepted widely, wildtype or recombinant, single or multiple strain/species. Multi-center and longer-term trials that are coordinated in their research methodology and analysis of data need to be undertaken to compare the effects of variable factors such as genetic susceptibility to the disease and genetic acceptance to the biotic therapy suggested here. In the interim, clinicians and researchers alike should follow ongoing clinical trials such as NCT05110703 that investigates the effect of a daily prebiotic fiber meal replacement shake on the quality of life in T2D patients. In addition, trials such as NCT04089280 go a step beyond and aim to elucidate the effect of an 8-strain multispecies probiotic in their capacity to reduce metformin-induced GI adverse effects. Finally, NCT04769687 is perhaps the most promising trial that will attempt to investigate the effect of a twice-daily 8-strain multispecies, oligofructose and Raftiline HP synbiotic on a variety of parameters among T2D plus CKD patients, with the primary outcome of CRP levels, followed by inflammatory cytokines, circulating monocytes, microbial metabolome, membrane permeability, bacterial translation, quality of life and frailty.

## Data Availability Statement

The original contributions presented in the study are included in the article/supplementary material, further inquiries can be directed to the corresponding author/s.

## Author Contributions

AC designed the study, critically supervised the project, revised and reviewed the manuscript, and initially screened studies. RS performed the database search, references extraction, methodology section draft and flowchart, and reviewed and edited the manuscript. BA screened the study abstracts and full-texts. PP and RK further critically screened full-texts, extracted, and analyzed the data, updated the search, wrote majority of the manuscript, generated most of the tables, and further edited the manuscript. MA wrote parts of the manuscript and generated a table. MS and RU reviewed and edited the manuscript, and generated one figure. All authors have read and agreed to the published version of the manuscript.

## Conflict of Interest

The authors declare that the research was conducted in the absence of any commercial or financial relationships that could be construed as a potential conflict of interest.

## Publisher’s Note

All claims expressed in this article are solely those of the authors and do not necessarily represent those of their affiliated organizations, or those of the publisher, the editors and the reviewers. Any product that may be evaluated in this article, or claim that may be made by its manufacturer, is not guaranteed or endorsed by the publisher.
